# Bevacizumab, tislelizumab and nab-paclitaxel for previously untreated metastatic triple-negative breast cancer: a phase II trial

**DOI:** 10.1136/jitc-2024-011314

**Published:** 2025-04-08

**Authors:** Meiting Chen, Riqing Huang, Qixiang Rong, Wei Yang, Xiujiao Shen, Qi Sun, Ditian Shu, Kuikui Jiang, Cong Xue, Jing Peng, Xin An, Haifeng Li, Fei Xu, Yanxia Shi

**Affiliations:** 1 Department of Medical Oncology, Sun Yat-sen University Cancer Center, Guangzhou, Guangdong, People's Republic of China; 2 State Key Laboratory of Oncology in South China, Sun Yat-sen University Cancer Center, Guangzhou, Guangdong, People's Republic of China; 3 Guangdong Provincial Clinical Research Center for Cancer, Guangzhou, Guangdong, People's Republic of China; 4 Department of Pathology, Sun Yat-sen University Cancer Center, Guangzhou, Guangdong, People's Republic of China

**Keywords:** Breast Cancer, Immunotherapy, Biomarker

## Abstract

**Background:**

Optimal first-line therapy for metastatic triple-negative breast cancer (mTNBC) varied in different situations. This phase II trial explores the efficacy and safety of combination regimens with **be**vacizumab, **ti**slelizumab and **na**b-paclitaxel (BETINA) in first-line setting for mTNBC.

**Methods:**

Patients with previously untreated advanced TNBC received tislelizumab 200 mg and bevacizumab on day 1 and nab-paclitaxel 125 mg/m^2^ on day 1, day 8 in 3-week cycles. Patients were randomized to bevacizumab 7.5 mg/kg or 15 mg/kg. The primary endpoint was investigator-assessed objective response rate (ORR) per Response Evaluation Criteria in Solid Tumors V.1.1. Secondary endpoints included progression-free survival (PFS), overall survival (OS), and safety. The trial was registered at the Chinese Clinical Trial Registry (No. ChiCTR2200058567).

**Results:**

30 female patients were enrolled from March 11, 2021 to February 5, 2024. Nine patients receiving bevacizumab 15 mg/kg experienced significantly higher hypertension rates versus 7.5 mg/kg (55.5% vs 0%), prompting subsequent enrollment of 12 additional patients at 7.5 mg/kg. By November 30, 2024, the ORR was 73.3% and the disease control rate was 90.0%, while the median PFS was 6.0 months and the median OS was 19.8 months. No new safety signal was reported. Common treatment-related adverse events (AEs) included peripheral sensory neuropathy (83.3%), dyspepsia (70.0%), anemia (70.0%), leukocytopenia (66.7%), and pruritus (53.3%). Hypothyroidism (30.0%) was the most frequent immune-related AE. Biomarker analysis indicated that lower baseline interleukin (IL)-1α was associated with poor survival, while IL-2, vascular endothelial growth factor-A and insulin-like growth factor binding protein-7 levels significantly decreased at progression. RNA sequencing highlighted the enrichment of the fatty acid metabolism pathway in poor responders.

**Conclusions:**

BETINA study demonstrated promising efficacy and favorable tolerance in treating patients with mTNBC with bevacizumab with tislelizumab and nab-paclitaxel.

**WHAT IS ALREADY KNOWN ON THIS TOPIC**

- The optimal first-line chemotherapy regimen for metastatic triple-negative breast cancer (mTNBC) remains a significant challenge, particularly for programmed death-ligand 1 (PD-L1) negative patients, who constitute approximately 70% of the population and derive limited benefit from immunotherapy. Anti-angiogenic agents, such as bevacizumab, have shown potential to synergize with immune checkpoint inhibitors (ICIs) by enhancing the efficacy of programmed cell death protein-1 (PD-1)/PD-L1 blockade across various solid tumors. However, the combination of bevacizumab with ICIs and chemotherapy in the first-line setting for mTNBC has not been extensively explored.

**WHAT THIS STUDY ADDS**

- This phase II trial evaluates the efficacy and safety of combining **be**vacizumab with **ti**slelizumab (an anti-PD-1 antibody) and **na**b-paclitaxel (BETINA regimen) in previously untreated patients with mTNBC. The study demonstrates a promising objective response rate of 73.3% and favorable tolerability, with a median progression-free survival (PFS) of 6.0 months and overall survival of 19.8 months. Additionally, the study identifies potential biomarkers, such as pretreatment interleukin (IL)-1α levels, which may predict PFS, and highlights the role of serum vascular endothelial growth factor-A, IL-2, and insulin-like growth factor binding protein-7 in monitoring treatment response and resistance.

**HOW THIS STUDY MIGHT AFFECT RESEARCH, PRACTICE OR POLICY**

- The BETINA regimen offers a viable first-line treatment option for mTNBC, particularly for patients without liver metastasis, who exhibited higher response rates and longer PFS. The findings suggest that lower doses of bevacizumab (7.5 mg/kg) may be equally effective and better tolerated compared with higher doses, potentially reducing toxicity. Furthermore, the identification of IL-1α as a predictive biomarker could guide personalized treatment strategies, while the observed changes in serum cytokine levels may provide insights into mechanisms of resistance, informing future therapeutic approaches.

## Introduction

 Triple-negative breast cancer (TNBC) represented a heterogeneous group of cancers, some of which were associated with an aggressive course and a dismal prognosis.^1^ The addition of immune checkpoint inhibitors (ICIs) to standard chemotherapy as first-line therapy has demonstrated significant improvement in progression-free survival (PFS) and overall survival (OS).^2^,^4^ However, approximately 70% of programmed death-ligand 1 (PD-L1) negative patients do not benefit from the addition of immunotherapy.^5^ Therefore, exploring strategies to expand the application of immunotherapy to a broader population is of great importance.

The combination of chemotherapy with AKT pathway inhibitors, such as ipatasertib and capivasertib, failed to improve survival outcomes in TNBC.^6^ The addition of cobimetinib to atezolizumab and a taxane, targeted on mitogen-activated protein kinase pathway, did not result in a statistically significant increase in the objective response rate (ORR) compared with chemotherapy alone.^7^ Therefore, the optimal partner for chemotherapy in TNBC remains a significant challenge. Anti-angiogenic agents have been demonstrated to synergize programmed death-1 (PD-1) blockade, enhancing the efficacy of anti-PD-1 and anti-PD-L1 antibodies across varied solid tumors. In the FUTURE-C-plus trial, the addition of famitinib to ICIs and chemotherapy showed certain benefits in the immunomodulatory subtype of TNBC in the first-line setting.^8^ However, the classification of TNBC subtypes, which is based on the Fudan University Shanghai Cancer Center breast cancer panel, has not been widely adopted nationwide, posing challenges in identifying potential beneficiary populations in clinical practice.

Numerous clinical trials have demonstrated that the combination of bevacizumab of chemotherapy significantly improves PFS in patients with metastatic human epidermal growth factor receptor (HER)-2 negative breast cancer.^9^,^12^ Nonetheless, the therapeutic outcomes of combining bevacizumab with ICIs and chemotherapy remain unexplored. A phase II study investigating the efficacy of camerelizumab in combination with eribulin, and apatinib in pretreated metastatic TNBC (mTNBC) reported an ORR of 37% and a median PFS (mPFS) of 8.1 months.^13^ The triad rationale of integrating anti-angiogenic agents, PD-1 antibodies, and chemotherapy is compelling, and the incorporation of bevacizumab into immunotherapy and chemotherapy regimens has not been previously documented in the first-line setting. Consequently, we initiated the **be**vacizumab, **ti**slelizumab and **na**b-paclitaxel (BETINA) study, a prospective, phase II, single-arm trial, to evaluate the efficacy and safety of combining bevacizumab, tislelizumab and nab-paclitaxel, and to identify the potential biomarkers predictive of treatment response.

## Methods

### Study design and participants

Eligible patients were enrolled at the Sun Yat-sen University Cancer Center (SYSUCC) from March 11, 2021 to February 5, 2024 in our study. The primary inclusion criteria were as follows: (1) histologically confirmed TNBC with inoperable, locally advanced, or metastatic disease at enrollment. HER2-negative, estrogen receptor-negative, and progesterone receptor-negative status was confirmed by the pathology department of SYSUCC prior to enrollment, in accordance with guidelines of the American Society of Clinical Oncology and the College of American Pathologists; (2) aged 18–75 at screening; (3) scored 0–2 on Eastern Cooperative Oncology Group (ECOG); (4) life expectancy ≥6 months; (5) had measurable disease per Response Evaluation Criteria in Solid Tumors (RECIST) V.1.1; (6) completion of radiotherapy or prior curative chemotherapy at least 12 months before enrollment; (7) asymptomatic central nervous system (CNS) metastases were permitted; (8) adequate organ and hematological function, the results of blood test at baseline met all the following criteria: hemoglobin ≥85 g/L, absolute neutrophil count ≥1.5×10^9^/L, platelet ≥75×10^9^ /L, alanine aminotransferase (ALT) and aspartate aminotransferase (AST) ≤2.5×upper limit of normal (ULN) or ≤5×ULN for patients with hepatic metastases, and creatinine ≤1×ULN or creatinine clearance ≥50 mL/min, as calculated by the Cockcroft-Gault equation. Key exclusion criteria included: (1) untreated or symptomatic CNS metastases; (2) administration of immunosuppressive agents, systemic corticosteroids, or absorbable local corticosteroids (>10 mg/day prednisone or other therapeutic corticosteroid) within 2 weeks prior to enrollment; (3) serious infection or autoimmune disease; (4) prior treatment with ICIs.

The trial was done in accordance with Good Clinical Practice guidelines and the Declaration of Helsinki. Protocol approval was obtained from ethics committees of the SYSUCC. All patients provided written informed consent. The clinical trial was registered at the Chinese Clinical Trial Registry (No. ChiCTR2200058567).

### Procedures

Patients were randomized into two subgroups, receiving different doses of bevacizumab: 7.5 mg/kg or 15 mg/kg, administered intravenously every 3 weeks. All patients were treated with tislelizumab, an anti-PD-1 antibody, at a fixed dose of 200 mg intravenously, and nab-paclitaxel 125 mg/m^2^ on day 1, day 8 intravenously, in 3-week cycles until disease progression, intolerable toxicity, or death. Dose adjustments or discontinuation were permitted for patients intolerant to nab-paclitaxel or bevacizumab, whereas dose adjustments were not allowed for tislelizumab. Dose reductions were planned in the events of grade 3 and 4 toxicities as per Common Terminology Criteria for Adverse Events (CTCAE) V.5.0. Additionally, dose reductions were scheduled if chemotherapy was delayed for more than 7 days due to insufficient neutrophil or platelet count. A 25% reduction from the prior delivered dose was planned if the delay in the next chemotherapy cycle was between 8 and 14 days, while a 50% reduction was planned if the delay exceeded 14 days.

Treatment was continued until the occurrence of death, progressive disease (PD), unacceptable toxicity, loss to follow-up, or a decision to discontinue by either the patient or the investigator. Chemotherapy was discontinued in patients experiencing dose-limited toxicity. Patients who exhibited disease progression during the combination therapy were withdrawn from the study.

### Outcomes

The primary outcome was ORR, which was calculated as the proportion of patients achieving complete remission (CR) or partial remission (PR) evaluated using RECIST V.1.1. CT was routinely performed at baseline, every 6 weeks until disease progression. The secondary outcome included PFS, OS, disease control rate (DCR), clinical benefit rate (CBR), duration of response (DOR), and safety. PFS is defined as the time from randomization to tumor progression or death. OS was measured from the initiation of treatment until death. The duration of response (DOR) is defined as the time from the first evaluation of CR, PR, or stable disease (SD) to PD. DCR was calculated as the proportion of patients achieving CR, PR, or SD evaluated using RECIST V.1.1. CBR was defined as the percentage of patients who have achieved CR, PR or SD≥6 months evaluated using RECIST V.1.1.

The adverse events (AEs) were graded according to the CTCAE V.5.0. The relation of each AE with nab-paclitaxel, tislelizumab, or bevacizumab was considered possibly, probably, or likely related to treatment and estimated as the proportion of all toxicity-evaluable cycles in which toxicity was observed.

### Serum biomarker analysis

The exploratory outcome included dynamic serum cytokine concentration and serum levels of bevacizumab. Blood samples were collected prior to treatment initiation, before each subsequent infusion of bevacizumab every two treatment cycles, and at the end of treatment. Serum concentration of bevacizumab was quantified using validated enzyme-linked immunosorbent analysis (ELISA). Serum samples of all patients were analyzed using a commercially available Luminex multifactor detection liquid-phase chip (R&D system, USA) coupled with the Luminex xMAP technology, a multiplex bead-based assay system that enables the simultaneous detection of multiple analytes.^14^ A comprehensive panel of biomarkers was measured, including Th1 (interferon (IFN)-α, IFN-β, IFN-γ, interleukin (IL)-2, IL-2Rα), Th2 (IL-4, IL-6, IL-10), inflammatory cytokines (IL-1α, IL-1β), tumor angiogenesis markers (vascular endothelial growth factor (VEGF)-A, VEGF-C), insulin-like growth factor binding proteins (insulin-like growth factor binding protein (IGFBP)-1, IGFBP-2, IGFBP-3, IGFBP-4, IGFBP-6, IGFBP-7), chemokines [C-X-C chemokine ligand (CXCL)2, CXCL9, CXCL10, CXCL11, CXCL13] as well as matrix metalloproteinases (MMP)-7, MMP-10, IL-17, monocyte chemotactic protein (MCP)-1, Fibroblast growth factor (FGF)2, following the manufacturer’s instructions (Bio-Plex Pro Human Cytokine Assay, Bio-Rad Laboratories, USA).

### Immunohistochemistry

Partial or complete linear membrane staining of any intensity in malignant cells and any membrane and/or cytoplasmic staining of any intensity for lymphocytes and macrophages were scored. Only lymphocytes and macrophages directly related to the tumor tissue were included. The combined positive score (CPS) is presented as PD-L1 staining cells (tumor cells, lymphocytes, macrophages) divided by the total number of viable tumor cells multiplied by 100. The CPS score was evaluated at a magnification of 20×. A positive CPS score was ≥10, while a negative score was <10.

### RNA sequencing

Total RNA was extracted from tumor samples. After quality control of RNA amount, purity, and integrity, complementary DNA library with 300±50 bp size was generated from~1 µg of total RNA. Then the library was sequenced on an Illumina NovaSeq 6000 using 2×150 bp paired-end sequencing chemistry. Differentially expressed genes were defined as fold change >2 or fold change <0.5 and p<0.05, and then Gene Ontology and Kyoto Encyclopedia of Genes and Genomes pathway enrichment analyses were done. All services were provided by LC Biotech Corporation (Hangzhou, China).

### Statistical analysis

The sample size for this study was calculated using a single-stage phase II design. Based on a historical ORR of 20%, the combination regimen was anticipated to achieve a response rate of 70%. With a significance level (α) of 0.05, a power (β) of 0.2, and an estimated dropout rate of 10%, a total of 30 patients were required for enrollment. Efficacy and survival analyses were primarily conducted in the intention-to-treat (ITT) population, which included patients who received at least one dose of nab-paclitaxel, bevacizumab, and tislelizumab and had adequate baseline tumor assessments. Descriptive statistics were employed to summarize patient characteristics, treatment administration, antitumor activity, and safety, with results presented as medians and ranges. Categorical variables were compared using the χ^2^ test or Fisher’s exact test, as appropriate. OS and PFS were assessed using Kaplan-Meier analysis in GraphPad Prism V.9.01 (GraphPad Software, San Diego, California, USA), and R V.4.4.1 (The R Project for Statistical Computing, www.r-project.org). The median follow-up time was calculated using the reverse Kaplan-Meier method. A two-sided p-value of <0.05 was considered statistically significant.

## Results

### Patients and treatment

**Figure 1:**
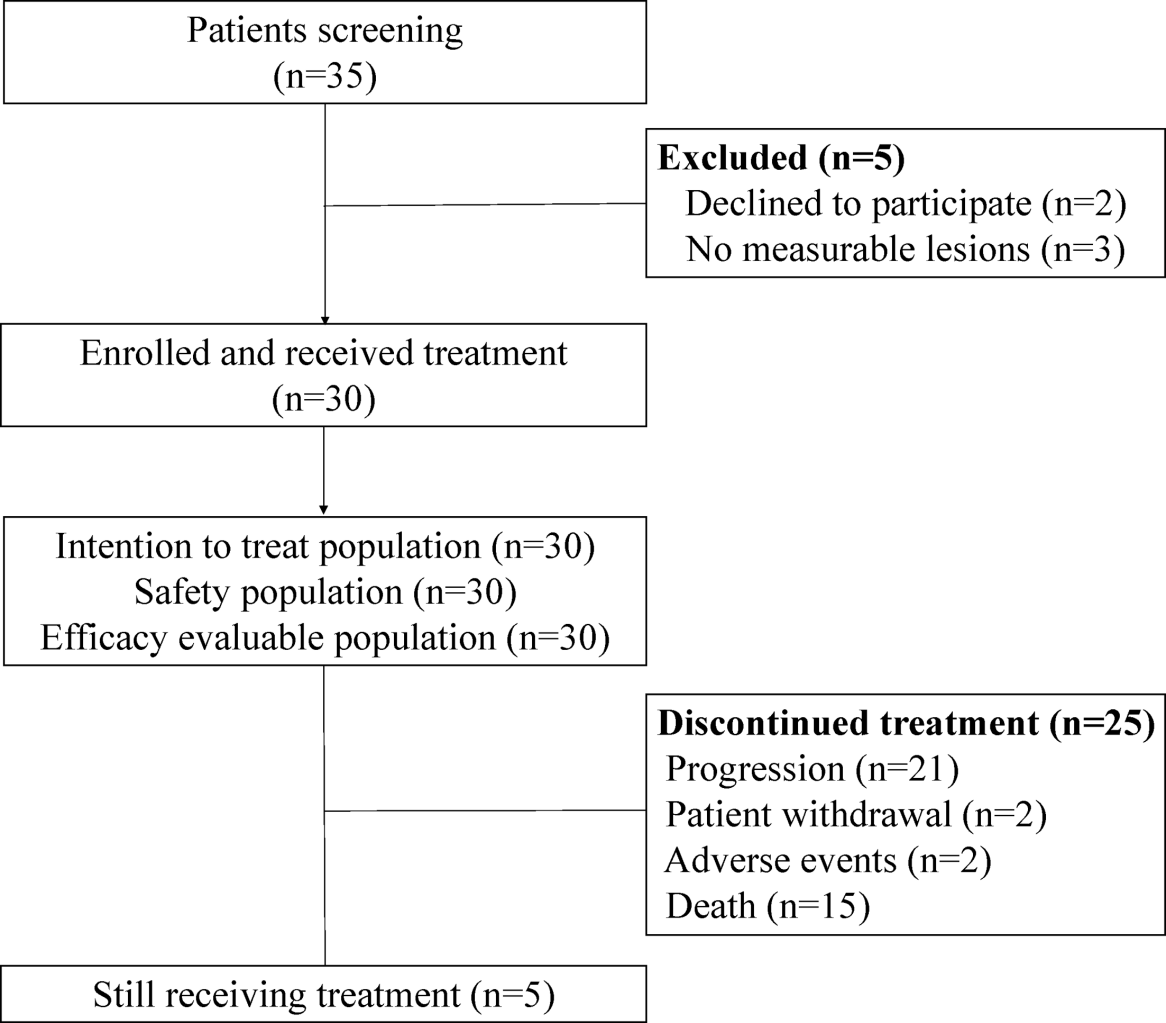
Trial profiles.

30 eligible patients enrolled in our study from March 11, 2021 to February 5, 2024. Safety and efficacy analyses were conducted for all participants, and the trial profile is illustrated in figure 1. As of the cut-off date of December 1, 2024, median follow-up time was 26.7 months (95% CI 20.6 to not applicable (NA) months). At the time of analysis, 15 (50.0%) of 30 patients had died, and 5 (16.7%) patients remained on treatment. The baseline characteristics were summarized in table 1. In our study, 70.0% patients had visceral metastases, and 23.3% patients had brain metastases.

**Table 1:** Baseline characters

	Dose of bevacizumab		
	**7.5 mg/kg (N=21**)	**15 mg/kg (N=9**)	**Overall (N=30**)
Age			
Mean (SD)	48.0 (11.3)	55.9 (9.68)	50.4 (11.3)
Median (Min, Max)	47.0 (30.0, 74.0)	53.0 (41.0, 69.0)	50.5 (30.0, 74.0)
ECOG			
0	1 (4.8%)	3 (33.3%)	4 (13.3%)
1	19 (90.5%)	5 (55.6%)	24 (80.0%)
2	1 (4.8%)	1 (11.1%)	2 (6.7%)
Menopausal status			
Premenopausal	15 (71.4%)	4 (44.4%)	19 (63.3%)
Postmenopausal	6 (28.6%)	5 (55.6%)	11 (36.7%)
PD-L1 expression			
CPS<10	7 (33.3%)	1 (11.1%)	8 (26.7%)
CPS≥10	2 (9.5%)	1 (11.1%)	3 (10.0%)
Unknown	12 (57.1%)	7 (77.8%)	19 (63.3%)
HER2 expression			
0	11 (52.4%)	3 (33.3%)	14 (46.7%)
1+	7 (33.3%)	2 (22.2%)	9 (30.0%)
2+ and FISH negative	3 (14.3%)	4 (44.4%)	7 (23.3%)
Disease status			
De novo metastasis	8 (38.1%)	2 (22.2%)	10 (33.3%)
Recurrent after surgery	13 (61.9%)	7 (77.8%)	20 (66.7%)
Previous taxane-based neo/adjuvant chemotherapy			
No	1 (4.8%)	1 (11.1%)	2 (6.7%)
Yes	12 (57.1%)	6 (66.7%)	18 (60.0%)
Previous anthracycline-based neo/adjuvant chemotherapy			
No	3 (14.3%)	2 (22.2%)	5 (16.7%)
Yes	10 (47.6%)	5 (55.6%)	15 (50.0%)
Number of metastases			
<3	14 (66.7%)	5 (55.6%)	19 (63.3%)
≥3	7 (33.3%)	4 (44.4%)	11 (36.7%)
Visceral metastasis			
No	8 (38.1%)	1 (11.1%)	9 (30.0%)
Yes	13 (61.9%)	8 (88.9%)	21 (70.0%)
Lymph node metastasis			
No	4 (19.0%)	1 (11.1%)	5 (16.7%)
Yes	17 (81.0%)	8 (88.9%)	25 (83.3%)
Lung metastasis			
No	13 (61.9%)	4 (44.4%)	17 (56.7%)
Yes	8 (38.1%)	5 (55.6%)	13 (43.3%)
Brain metastasis			
No	16 (76.2%)	7 (77.8%)	23 (76.7%)
Yes	5 (23.8%)	2 (22.2%)	7 (23.3%)
Liver metastasis			
No	14 (66.7%)	6 (66.7%)	20 (66.7%)
Yes	7 (33.3%)	3 (33.3%)	10 (33.3%)
Chest wall metastasis			
No	19 (90.5%)	8 (88.9%)	27 (90.0%)
Yes	2 (9.5%)	1 (11.1%)	3 (10.0%)
Bone metastasis			
No	11 (52.4%)	7 (77.8%)	18 (60.0%)
Yes	10 (47.6%)	2 (22.2%)	12 (40.0%)
Pleural metastasis			
No	20 (95.2%)	6 (66.7%)	26 (86.7%)
Yes	1 (4.8%)	3 (33.3%)	4 (13.3%)

CPScombined positive scoreECOGEastern Cooperative Oncology GroupFISHfluorescence in situ hybridizationHER2Human epidermal growth factor receptor 2PD-L1programmed death-ligand 1

### Efficacy

**Figure 2:**
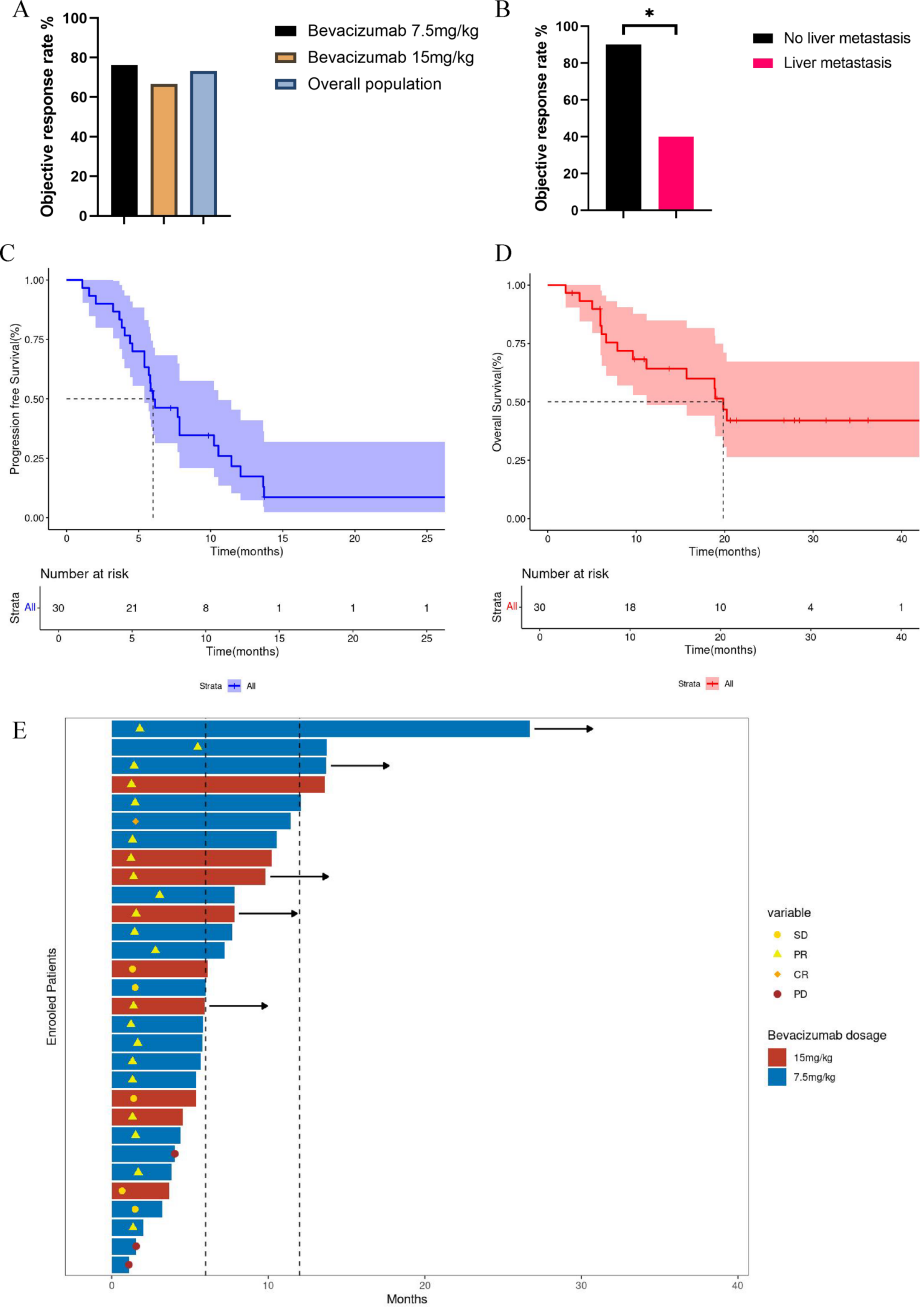
The ORR for patients receiving 7.5 mg/kg, 15 mg/kg bevacizumab and overall population (**A**). The ORR for patients with and without liver metastasis (**B**). The progression-free survival (**C**) and overall survival (**D**) for all patients. (**E**) The swimming plot for all enrolled patients receiving different doses of bevacizumab. CR, complete remission; ORR, objective response rate; PD, progressive disease; PR, partial remission; SD, stable disease.

In the ITT population, the ORR, CBR, and DCR were 73.3%, 86.7% and 90.0%, respectively. The ORR was 76.2% in the bevacizumab 7.5 mg/kg group and 66.7% in the bevacizumab 15 mg/kg group (figure 2A). Among seven patients with brain metastasis, the CNS ORR was 16.7%. Similar CBR and DCR were presented in the bevacizumab 7.5 mg/kg and 15 mg/kg group (table 2). Significantly higher ORR was observed in patients without liver metastasis (90.0% vs 40.0%, p=0.0072, figure 2B, table 2).

**Table 2:** Efficacy profiles

	Liver metastasis		Dose of bevacizumab		
	**No**(**N=20**)	**Yes**(**N=10**)	**7.5 mg/kg (N=21**)	**15 mg/kg (N=9**)	**Overall (N=30**)
Best tumor response					
CR	1 (5.0%)	0	1 (4.8%)	0	1 (3.3%)
PR	17 (85.0%)	4 (40.0%)	15 (71.4%)	6 (66.7%)	21 (70.0%)
SD	2 (10.0%)	3 (30.0%)	2 (9.5%)	3 (33.3%)	5 (16.7%)
PD	0 (0%)	3 (30.0%)	3 (14.3%)	0	3 (10.0%)
ORR	18 (90.0%)	4 (40.0%)	16 (76.2%)	6 (66.7%)	22 (73.3%)
CBR	20 (100%)	6 (60.0%)	17 (81.0%)	9 (100%)	26 (86.7%)
DCR	18 (90.0%)	7 (70.0%)	16 (76.2%)	9 (100%)	25 (83.3%)

CBRclinical benefit rateCRcomplete remissionDCRdisease control rateORRobjective response ratePDprogressive diseasePRpartial remissionSDstable disease

The mPFS was 6.0 months (95% CI 5.4 to 10.5) and the median OS was 19.8 months (95% CI 11.2 to NA) (figure 2C and D). The swimmer plot for all patients was shown in figure 2E. The analyses revealed no significant difference in PFS between patients receiving bevacizumab at 7.5 mg/kg and 15 mg/kg doses (mPFS 5.8 months vs 7.8 months, HR=1.08, 95% CI 0.46 to 2.55, p=0.86, online supplemental figure S1A). However, patients with liver metastases demonstrated significantly inferior PFS outcomes (mPFS 4.7 months vs 7.8 months, HR=2.79 95% CI 0.98 to 7.97, p*=*0.0073, online supplemental figure S1B). Similarly, patients with brain metastases showed markedly reduced PFS (mPFS 3.8 months vs 7.8 months, HR=5.44, 95% CI 1.13 to 26.2, p<0.0001, online supplemental figure S1C), with a CNS PFS of 3.67 months. No significant difference in PFS was observed between patients with de novo and recurrent disease (mPFS 6.0 months vs 6.8 months, HR=0.79, 95% CI 0.34 to 1.83, p*=*0.94, online supplemental figure S1D). Furthermore, the mPFS in patients with HER-2 low and HER-2 0 was 5.6 months and 7.7 months (HR=0.83, 95% CI 0.38 to 1.82, p=0.43, online supplemental figure S1E), respectively.

Patients with brain metastasis demonstrated significantly inferior OS outcomes (median OS 6.1 months vs not reached, HR=6.03, 95% CI 1.32 to 27.6, p<0.001, online supplemental figure S2A). Similarly, patients with liver metastasis showed a trend toward poorer survival, although this did not reach statistical significance (median OS 7.9 months vs 20.2, HR=2.61, 95% CI 0.72 to 9.17, p*=*0.056, online supplemental figure S2B). The analysis of bevacizumab dosing regimens revealed no significant difference in OS between the 7.5 mg/kg and 15 mg/kg groups (20.2 months vs 11.2 months, HR=0.62, 95% CI 0.19 to 2.04, p=0.577, online supplemental figure S2C). Furthermore, similar OS outcomes were observed between patients with de novo and recurrent disease (online supplemental figure S2D).

A notable case is illustrated in online supplemental figure S3. Patient #28, an adult female, presented with inoperable disease in the left breast accompanied by axillary and cervical lymph node metastases, initially staged as cT4N3M1. Following five cycles of combination therapy with bevacizumab, tislelizumab, and nab-paclitaxel, the patient achieved a best response of PR. After comprehensive evaluation by the multidisciplinary team in SYSUCC, the patient opted to withdraw from our study and underwent modified radical mastectomy. Histopathological examination confirmed pathologic CR (pCR) in both the breast and axillary lymph nodes, with post-treatment staging of ypT0N0. Subsequent adjuvant radiotherapy was administered following surgery. Follow-up imaging studies, including ultrasound and CT scans, revealed no detectable abnormalities in the bilateral cervical lymph nodes. At the most recent follow-up, she was maintained on capecitabine and remained disease-free for 7.3 months.

### Safety

In the initial safety analysis of 18 patients, conducted in accordance with Institutional Review Board requirements of SYSUCC, we observed a significantly higher incidence of hypertension in the bevacizumab 15 mg/kg group compared with the bevacizumab 7.5 mg/kg group (55.5% vs 0%, p=0.029). Notably, one serious adverse event (SAE) of hypertension was reported in the 15 mg/kg group. Additionally, the incidence of proteinuria was elevated in the 15 mg/kg group. Detailed safety profiles are presented in online supplemental table 1. Based on these safety findings, we amended the study protocol and subsequently and enrolled next 12 patients in the bevacizumab 7.5 mg/kg group. Consequently, the final cohort comprised nine patients receiving bevacizumab 15 mg/kg and 21 patients treated with bevacizumab 7.5 mg/kg.

No new safety signal was reported. Among the cohort, six patients experienced treatment-related AEs. Disease progression led to SAEs in two patients. The reported SAEs included two case of sepsis shock, one case of severe hypertension, and three severe immunotherapy-related AEs (irAEs), including hypothyroidism, myocarditis, and hepatitis. The common hematological grade 3/4 AEs were leukopenia and neutropenia. Among non-hematological AEs, peripheral sensory neuropathy, alopecia, and dyspepsia were most commonly observed (table 3). Hypothyroidism emerged as the most prevalent irAE. Notably, patients who developed irAEs demonstrated a trend toward longer PFS compared with those without irAEs, although this difference did not reach statistical significance (mPFS 10.2 months vs 5.6 months, HR=0.53, 95% CI 0.24 to 1.17, p=0.10, online supplemental figure S1F).

**Table 3:** Treatment-related adverse events

	Dose of bevacizumab					
	**7.5** **mg/kg**(**N=21**)		**15 mg/kg (N=9**)		**Overall (N=30**)	
	Any grade	Grade 3/4	Any grade	Grade 3/4	Any grade	Grade 3/4
Any AE	21 (100%)	9 (42.9%)	9 (100%)	3 (33.3%)	30 (100%)	12 (40.0%)
SAE	4 (19.0%)	4 (19.0%)	2 (22.2%)	2 (22.2%)	6 (20.0%)	6 (20.0%)
Leukopenia	15 (71.4%)	6 (28.6%)	5 (55.6%)	1 (11.1%)	20 (66.7%)	7 (23.3%)
Neutropenia	13 (61.9%)	5 (23.8%)	5 (55.6%)	1 (11.1%)	18 (60.0%)	6 (20.0%)
Febrile neutropenia	0	2 (9.5%)	0	0	0	2 (6.7%)
Anemia	16 (76.2%)	0	5 (55.6%)	0	21 (70.0%)	0
Thrombocytopenia	4 (19.0%)	2 (9.5%)	0	0	4 (13.3%)	2 (6.7%)
Peripheral sensory neuropathy	17 (81.0%)	0	8 (88.9%)	0	25 (83.3%)	0
Dyspepsia	15 (71.4%)	0	6 (66.7%)	0	21 (70.0%)	0
Alopecia	14 (66.7%)	0	5 (55.6%)	0	19 (63.3%)	0
Dizziness	14 (66.7%)	0	2 (22.2%)	0	16 (53.3%)	0
Pruritus	12 (57.1%)	0	4 (44.4%)	0	16 (53.3%)	0
Nausea	11 (52.4%)	0	4 (44.4%)	0	15 (50.0%)	0
Fatigue	10 (47.6%)	0	3 (33.3%)	0	13 (43.3%)	0
Diarrhea	10 (47.6%)	0	2 (22.2%)	0	12 (40.0%)	0
Constipation	9 (42.9%)	0	2 (22.2%)	0	11 (36.7%)	0
Abdominal pain	9 (42.9%)	0	2 (22.2%)	0	11 (36.7%)	0
Oral ulcer	6 (28.6%)	0	5 (55.6%)	0	11 (36.7%)	0
Myalgia	7 (33.3%)	0	3 (33.3%)	0	10 (33.3%)	0
Rash	6 (28.6%)	0	3 (33.3%)	0	9 (30.0%)	0
Arthralgia	7 (33.3%)	0	2 (22.2%)	0	9 (30.0%)	0
Vomit	6 (28.6%)	0	3 (33.3%)	0	9 (30.0%)	0
Hypertension	2 (9.5%)	0	5 (55.5%)	1 (11.1%)	7 (23.3%)	1 (3.3%)
Epistaxis	3 (14.3%)	0	0	0	3 (10.0%)	0
ALT elevation	7 (33.3%)	2 (9.5%)	4 (44.4%)	0	11 (36.7%)	2 (6.7%)
AST elevation	8 (38.1%)	2 (9.5%)	4 (44.4%)	0	12 (40.0%)	2 (6.7%)
Serum creatinine elevation	2 (9.5%)	0	1 (11.1%)	0	3 (10.0%)	0
Serum total bilirubin elevation	3 (14.3%)	0	0	0	3 (10.0%)	0
Proteinuria	4 (19.0%)	0	0	0	4 (13.3%)	0
irAE	Any grade	Grade 3/4	Any grade	Grade 3/4	Any grade	Grade 3/4
Any irAE	8 (38.1%)	2 (9.5%)	4 (44.4%)	1 (11.1%)	12 (40.0%)	3 (10.0%)
Hypothyroidism	5 (23.8%)	0	4 (44.4%)	1 (11.1%)	9 (30.0%)	1 (3.3%)
Hyperthyroidism	4 (19.0%)	0	1 (11.1%)	0	5 (16.7%)	0
Adrenal cortical insufficiency	2 (9.5%)	0	0	0	2 (6.7%)	0
Myocarditis	1 (4.8%)	1 (4.8%)	0	0	1 (3.3%)	1 (3.3%)
Hepatitis	1 (4.8%)	1 (4.8%)	0	0	1 (3.3%)	1 (3.3%)
Rash	1 (4.8%)	0	0	0	1 (3.3%)	0

AEadverse eventALTalanine aminotransferaseASTaspartate aminotransferaseirAEimmunotherapy-related AESAEserious adverse event

### Univariate and multivariate analysis on PFS and OS

**Figure 3:**
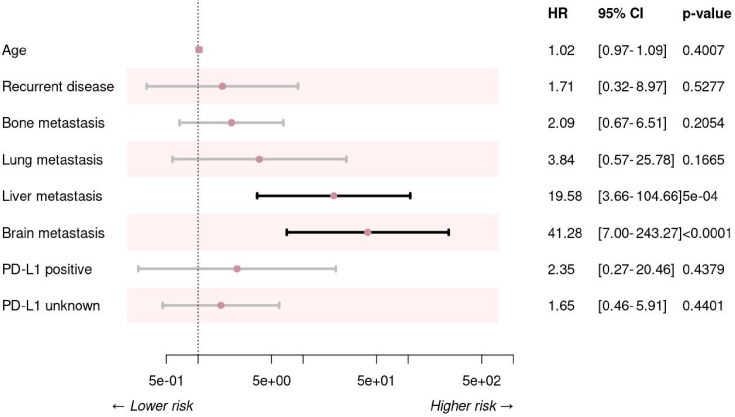
The forest plot of multivariate analysis for progression-free survival. PD-L1, programmed death-ligand 1.

Univariate analysis identified several clinical factors significantly associated with poor PFS, including brain, liver and bone metastasis. Subsequent forward conditional Cox regression analysis revealed that liver and brain metastases remained independent negative prognostic factors for PFS. Comprehensive details of these prognostic factors are provided in online supplemental table 2, with the corresponding multivariate analysis forest plot for PFS shown in figure 3.

In terms of OS, univariate analysis revealed that brain and bone metastasis were correlated with worse outcomes. Multivariate analysis further established brain, lung, and liver metastases as significant independent predictors of inferior OS. These findings are detailed in online supplemental table 3. The predictive accuracy of the nomograms for both PFS and OS was validated and illustrated in online supplemental figure S4A and B, respectively.

### Potential biomarkers

Since PD-L1 assessment was not required by the study protocol, PD-L1 (22C3) testing was performed in only 11 patients from the ITT population, based on tissue availability and reimbursement eligibility. PD-L1 positivity was defined as a CPS≥10. The swimming plot based on PD-L1 expression was shown in online supplemental figure S4. In our study, the mPFS for PD-L1 positive, PD-L1 negative, and unknown patients was 7.8 months, 10.5 months, and 5.8 months, respectively (online supplemental figure S4B). In addition, it was not reached in PD-L1 negative patients, while it was 19.6 months and 18.9 months for PD-L1 positive and PD-L1 unknown patients, respectively (online supplemental figure S4C).

**Figure 4:**
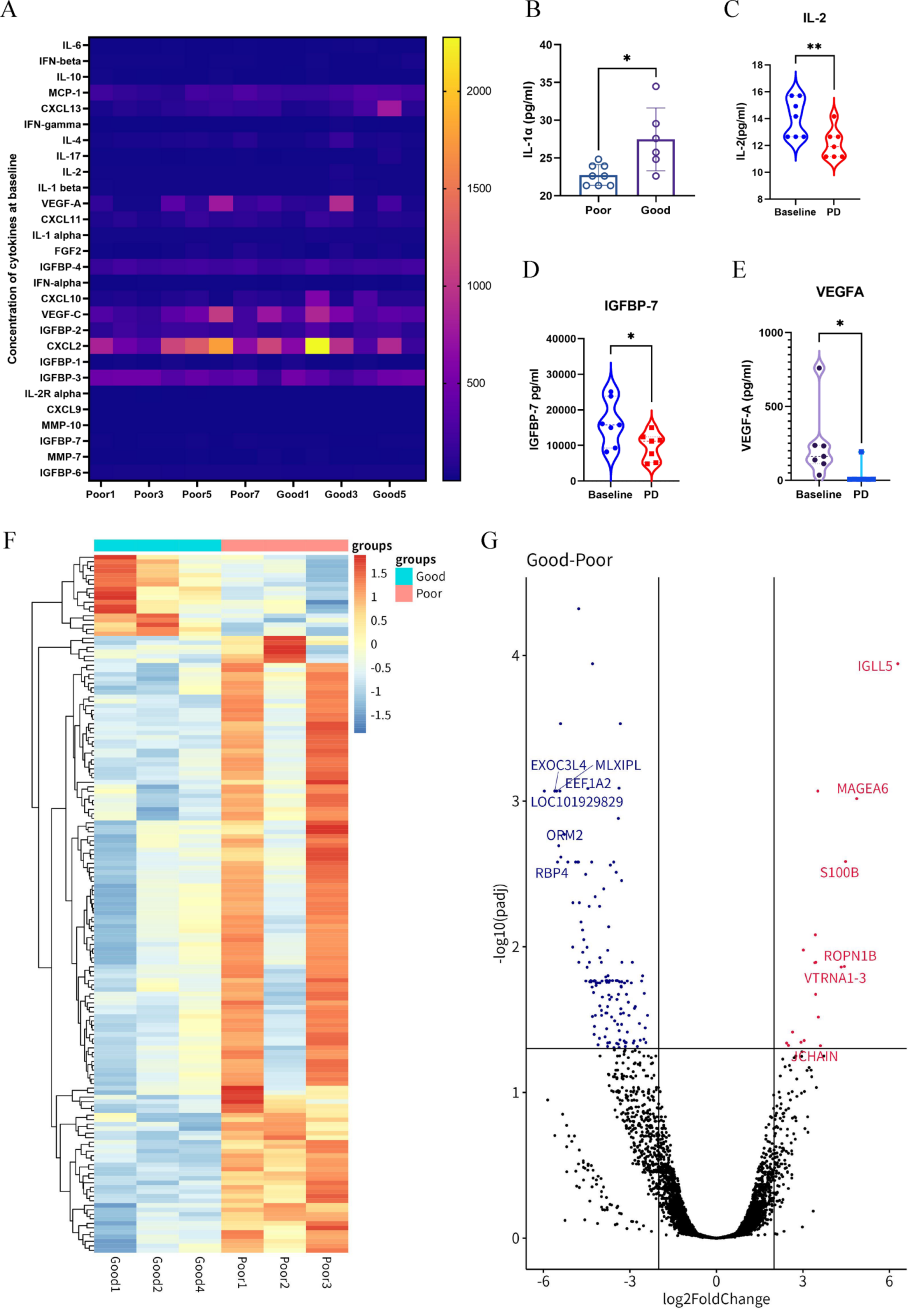
(**A**) The overview of baseline serum cytokine concentration. (**B**) The comparison of serum IL-1α concentration in patients with PFS≥6 months and those with PFS<6 months. The comparison of serum IL-2 (**C**), IGFBP-7 (**D**), VEGF-A (**E**) in baseline and at disease progression. The cluster analysis (**F**) and volcano plot (**G**) of RNA sequencing in patients with PFS≥6 months (good responders) and those with PFS<6 months (poor responders). CXCL, C-X-C chemokine ligand; MMP, matrix metalloproteinases; MCP, monocyte chemotactic protein; FGF, fibroblast growth factor; IFN, interferon; IGFBP, insulin-like growth factor binding protein; IL, interleukin; PD, progressive disease; PFS, progression-free survival; VEGF, vascular endothelial growth factor.

For genomic and serum biomarker analyses, patients were stratified into good responders (PFS>6 months) and poor responders (PFS≤6 months). Pretreatment serum samples from 14 patients (6 good responders and 8 poor responders) were analyzed for 28 cytokines, with paired baseline and end-of-treatment samples available for 7 patients. Notably, good responders demonstrated significantly higher baseline IL-1α levels compared with poor responders (IL-1α level: 27.475±4.169 pg/mL vs 22.758±1.364 pg/mL, p=0.0105, figure 4A and B). It revealed that significant decreases in serum IL-2, IGFBP-7, VEGF-A concentration at progression compared with baseline levels (IL-2 level: 4.067±1.425 pg/mL vs 12.127±1.119 pg/mL, p=0.0151; IGFBP-7: 9.688±3.857 ng/mL vs 16.190±6.467 ng/mL, p=0.041; VEGF-A: 33.853±70.017 pg/mL vs 239.976±239.850 pg/mL, p=0.049) (figure 4C, D and E). Although not statistically significant, good responders showed a trend toward higher serum bevacizumab concentrations compared with poor responders (bevacizumab concentration: 0.997±0.500 µg/mL vs 0.601±0.481 µg/mL, p=0.093) (online supplemental figure S6A), with lower concentration observed at progression compared with during active bevacizumab therapy (bevacizumab concentration: 0.185±0.262 µg/mL vs 1.050±0.457 µg/mL, p=0.108) (online supplemental figure S6B).

RNA sequencing analysis was conducted on pretreatment tumor specimens from four good responders and four poor responders. Differential expression analysis identified 18 upregulated and 117 downregulated genes in good responders compared with poor responders (figure 4F and G). Gene set enrichment analysis (GSEA) analysis revealed upregulation of IFN-γ response pathways and downregulation of fatty acid metabolism pathways in good responders (online supplemental figure S6C,D and E).

## Discussion

In our study, we evaluated the safety and efficacy profiles of bevacizumab, tislelizumab and nab-paclitaxel as first-line therapy of mTNBC. Comparable ORR, PFS and OS were observed between patients receiving bevacizumab at 7.5 mg/kg and those receiving 15 mg/kg, indicating that a lower dose of bevacizumab remains effective in TNBC when combined with immunotherapy. Patients without liver metastasis exhibited higher ORR, superior PFS, and OS compared with those with liver metastasis. Pretreatment IL-1α might serve as a potential biomarker for predicting PFS, while monitoring serum IGFBP-1, IL-2, VEGF-A and bevacizumab concentration could aid in identifying potential resistance mechanisms. RNA sequencing revealed upregulation of IL-6 signaling and downregulation of fatty acid metabolism pathways in patients with a PFS exceeding 6 months compared to those with shorter PFS.

Clinical guidelines and trials indicate that the optimal bevacizumab dosage for metastatic breast cancer is either 10 mg/kg every two weeks or 15 mg/kg every three weeks. Studies such as E2100, Ribbon-1, Ribbon-2, ATHENA, and NEWBEAT have consistently used these dosages,^911 15^,^17^ with the AVADO trial demonstrating superior mPFS for the 15 mg/kg q3w compared with 7.5 mg/kg when combined with docetaxel.^18^ However, higher AEs, including hypertension, proteinuria, and bleeding, were observed with the 15 mg/kg dose.^18^ The AVADO study reported that the incidence of hypertension was 4.5% in patients receiving bevacizumab 15 mg/kg compared with 0.8% in those receiving 7.5 mg/kg.^18^ A real-world analysis from the multicenter AVANTI study, involving over 2,000 patients, revealed that bevacizumab-related AEs occurred in 30% of cases.^19^ Notably, AEs such as epistaxis and proteinuria were more frequent in the 15 mg/kg group, although this group also showed a slightly longer PFS compared with the 7.5 mg/kg group.^20^ In the ATHENA study, which included 585 TNBC cases treated with bevacizumab at 10 mg/kg every 2 weeks or 15 mg/kg every 3 weeks, grade 3/4 hypertension was reported in 5% of patients, and arterial thromboembolic events occurred in 4%.^11^ Similarly, the NEWBEAT study, which evaluated nivolumab plus bevacizumab and paclitaxel in HER2-negative breast cancer patients, found that the incidence of any-grade hypertension was as high as 30%, with 14% being grade 3/4.^17^ These findings suggest that the 15 mg/kg dose of bevacizumab may increase toxicity, particularly when combined with immunotherapy. In our study, hypertension was more frequently observed in patients treated with bevacizumab 15 mg/kg, aligning with results from previous studies.^11 17 18^ Furthermore, several meta-analyses have confirmed that bevacizumab 15 mg/kg is associated with a higher incidence of AEs, especially for grade ≥3 AEs.^21^ Both prospective and real-world studies in lung cancer and ovarian cancer have demonstrated that bevacizumab 7.5 mg/kg is not inferior to bevacizumab 15 mg/kg, especially in elder patients and the Asian population.^22 23^ Given that tislelizumab is administered at 200 mg q3w, we chose bevacizumab 15 mg/kg q3w to align treatment schedules. Additionally, since the optimal bevacizumab dose in combination with ICIs remains undefined, we designed two subgroups to explore the efficacy and safety of 7.5 mg/kg and 15 mg/kg q3w. This approach aims to identify the most effective and tolerable dose for this combination therapy. It was indicated that bevacizumab 7.5 mg/kg might be a cost-effective option in the immunotherapy era.

The E1193 study demonstrated that doxorubicin and paclitaxel have equivalent efficacy, but paclitaxel is better tolerated with a lower risk of cardiotoxicity.^24^ The phase 2 TONIC trial highlighted that doxorubicin creates a more favorable tumor microenvironment, enhancing response to nivolumab in mTNBC with an ORR of 35%.^25^ Additionally, a phase I study in patients with anthracycline-naïve mTNBC treated with pembrolizumab plus doxorubicin reported an ORR of 67% and an mPFS of 5.2 months.^26^ However, anthracyclines are limited by cardiotoxicity, particularly at cumulative doses, and data from large-scale, phase III trials combining anthracyclines with immunotherapy remain scarce. In contrast, taxanes are safer for long-term use, with weekly dosing schedules being well-tolerated. Robust evidence from phase III trials, such as KEYNOTE 355, IMpassion 130, and TORCHLIGHT, supports the synergy of taxanes with immunotherapy in PD-L1-positive TNBC.^2 3 27^ Therefore, we opted for taxane combined with immune-checkpoint inhibitors over anthracycline-based regimens.

In the immunotherapy era, studies like IMpassion130 and TORCHLIGHT showed significant clinical benefits when nab-paclitaxel was combined with ICIs, unlike paclitaxel in IMpassion131.^3 27 28^ Given these findings, nab-paclitaxel appears optimal for immunotherapy combinations. Generally, clinical guidelines and trials support nab-paclitaxel monotherapy at 260 mg/m^2^ q3w or 100–150 mg/m^2^ weekly.^29^,^31^ A randomized multicenter study demonstrated superior PFS and safety with weekly nab-paclitaxel (100–150 mg/m^2^) compared with docetaxel, with lower rates of neutropenia than the 300 mg/m^2^ q3w regimen.^30^ However, peripheral neuropathy was more frequent at 150 mg/m^2^ weekly.^30 31^ Balancing efficacy and toxicity, we chose weekly nab-paclitaxel at a safer dose. Considering tolerance in the Chinese population, we referenced the TORCHLIGHT study, which used nab-paclitaxel at 125 mg/m^2^ on days 1 and 8 of a 21-day cycle.^3^ Thus, we selected nab-paclitaxel 125 mg/m^2^ day 1 and 8 as a clinically validated and safe dose in this setting.

Several first-line taxane-based trials reported an ORR of 34–49%, with mPFS of 4–6 months and median OS ranging from 12 to 18 months in the overall population.^2429^,^32^ However, some trials did not provide TNBC subgroup data.^24 29^ The TNT study, focusing on docetaxel versus carboplatin in TNBC, showed an ORR of 34%, PFS of 4.4 months, and OS of 12 months for docetaxel-treated patients.^32^ Previous studies, including E2100, AVADO, and Ribbon-1, reported ORRs of 51.3–64.1% for first-line taxanes plus bevacizumab.^9 15 18^ TNBC subgroup analyses from these phase III studies showed mPFS in the taxane control arm ranged from 4.7 to 6.0 months, while adding bevacizumab increased mPFS to 8.1–10.2 months.^33^ A meta-analysis of these trials demonstrated that adding bevacizumab to chemotherapy improved mPFS from 5.4 to 8.1 months in 621 patients with TNBC.^34^ In the CALGB 40502 study, mPFS for patients with TNBC treated with bevacizumab plus nab-paclitaxel and bevacizumab plus paclitaxel was 7.4 and 6.5 months, respectively.^35^ In conclusion, taxane-based therapies demonstrate variable efficacy in mTNBC, with the addition of bevacizumab showing improved PFS in some studies, highlighting its potential benefit in first-line treatment regimens.

In trials combining first-line taxanes with immunotherapy or targeted therapy, PFS outcomes in the control arms varied. The PAKT study reported an ORR of 28.8%, PFS of 4.2 months, and OS of 12.6 months for paclitaxel plus placebo.^36^ The CAPItello 290 study showed mPFS and OS of 5.1 and 18.0 months, respectively, for the same regimens.^37^ The COLET study reported lower efficacy, with an ORR of 20.9% and mPFS of 3.8 months.^7^ In contrast, the control arms of nab-paclitaxel in IMpassion 130 and TORCHLIGHT studies showed ORRs of 45.9–64.0%, mPFS of 5.5–6.9 months, and median OS of 17.6–23.5 months.^3^,^27^ While both nab-paclitaxel and paclitaxel in control arms demonstrate efficacy in TNBC treatment, nab-paclitaxel consistently shows higher ORRs, longer mPFS, and improved OS compared with paclitaxel across trials, highlighting its potential superiority in therapeutic outcomes. Our BETINA regimen achieved a comparable ORR, meeting the primary endpoint. However, the mPFS of 6.0 months remains suboptimal versus 7.2–8.4 months in larger trials like IMpassion130 and TORCHLIGHT. Given our smaller sample size, direct PFS comparisons are challenging. Further studies comparing triplet regimens with nab-paclitaxel-immunotherapy are warranted to optimize outcomes.

Preclinical studies have suggested that bevacizumab enhances the efficacy of ICIs in TNBC by promoting tumor infiltration of mature dendritic cells and effector T cells.^38^ Several prospective studies have explored the potential of combining anti-angiogenesis agents and ICIs. For instance, the combination of camrelizumab, apatinib, and eribulin in patients with heavily pretreated TNBC demonstrated an ORR of 37%, even in those with PD-L1-negative status or who had progressed after multiple lines of therapy, including ICIs.^13^ In a first-line setting, a phase II study reported that the combination of famitinib, camrelizumab, and nab-paclitaxel achieved a favorable ORR of 81.3% in advanced immunomodulatory TNBC.^8^ These findings suggest that triplet regimens may be particularly effective in highly selected patient populations. Similarly, our study indicated that the addition of anti-angiogenesis agents enhances antitumor activity, with a comparable ORR of 73.3%.

Both prospective and retrospective studies have demonstrated that PD-L1 expression is associated with survival outcomes in TNBC treated with immunotherapy.^3 4^ However, the predictive role of PD-L1 expression becomes less clear when anti-angiogenesis agents are combined with immunotherapy. For example, biomarker analysis in the NEWBEAT study did not reveal a correlation between tumor PD-L1 expression and the efficacy of triple therapy.^17^ In our cancer center, PD-L1 testing was not available prior to 2022 and was not eligible for reimbursement, leading to some patients not undergoing PD-L1 expression testing. Our study found no significant relationship between PD-L1 expression and PFS, consistent with the findings of the NEWBEAT study. This suggests that the addition of bevacizumab may partially alter immunoreactivity, potentially influencing the predictive value of PD-L1 expression.

In the biomarker analysis from the AVADO study, plasma vascular endothelial growth factor-A and VEGFR-2 emerged as potential predictive markers for the bevacizumab efficacy.^39^ However, the MERiDiAN study, which prospectively evaluated plasma VEGF-A as a predictive biomarker for bevacizumab efficacy in metastatic breast cancer, failed to identify a subset of patients who derived the greatest benefit from bevacizumab.^40^ In the neoadjuvant setting, patients who achieved a pCR after receiving bevacizumab exhibited significantly lower levels of VEGF-A, IFN-γ, tumor necrosis factor-α and IL-4 compared with those without pCR.^41^ The serum cytokine level is correlated with higher levels of cytotoxic T cells at the end of the therapy regimen, suggesting a link to bevacizumab treatment response.^41^ Nevertheless, the identification of reliable biomarkers to select patients who would benefit most from bevacizumab remains controversial, and biomarkers for predicting outcomes in patients receiving a combination of anti-angiogenesis agents, immunotherapy, and chemotherapy are still unknown. Some studies on metastatic colon rectal cancers have indicated that high bevacizumab concentrations are associated with reduced PFS and OS.^42^ Conversely, biomarker results from the phase II AVASTEM trial suggested that bevacizumab serum levels did not predict pathology remission and survival in the neoadjuvant setting.^43^ In our study, serum VEGF-A and IL-2 levels were significantly reduced at disease progression, consistent with previous findings. RNA sequencing results also revealed upregulation of the IL-6, IFN-γ, and IFN-α signaling pathways in good responders, aligning with the observed changes in serum cytokines. Additionally, serum bevacizumab concentrations decreased at disease progression, suggesting a potential marker for predicting resistance. Future research will focus on evaluating the predictive performance of the combination of serum VEGF-A and bevacizumab concentration in patients refractory to bevacizumab-based therapy.

In a phase II study evaluating the response to camrelizumab combined with apatinib and eribulin, a lipid proteomics model was shown to potentially predict ORR and PFS.^44^ To date, the role of circulating biomarkers in breast cancer progression has not been thoroughly investigated. IGFBP-7, a new member of a subgroup of the IGFBP-superfamily, is a secreted protein that binds to insulin, thereby inhibiting its anti-senescence and pro-growth effects.^45^ Preclinical research suggests that IGFBP-7 promotes acquired resistance to osimertinib in lung cancer.^46^ A prospective cohort study demonstrated that low levels of IGFBP-7 protein and messenger RNA expression were associated with less aggressive breast cancer characteristics.^47^ However, a nested case–control study found no association between preoperative IGFBP-7 and recurrence risk.^48^ Biomarker analysis in our study revealed that serum IGFBP-7 levels were lower at disease progression compared with baseline, suggesting a potential association with resistance. Additionally, RNA sequencing results highlighted differential expression of genes related to fatty acid metabolism and xenobiotic metabolism, which may interact with IGFBP-7. These findings underscore the need for further investigation into the role of IGFBP-7 in breast cancer.

The study has several limitations, including a moderate sample size and a single-arm, open-label study design. Although the absence of a comparator arm restricts the ability to draw definitive comparative conclusions, the results indicate that the triplet regimen of bevacizumab, tislelizumab, and nab-paclitaxel is tolerable and demonstrates clinical activity in certain patients with mTNBC. To further validate the efficacy of this triplet regimen as a first-line therapy for TNBC, a phase II randomized, double-blind study is currently in progress.

## Conclusion

The combination of bevacizumab, tislelizumab, and nab-paclitaxel demonstrated promising efficacy and favorable tolerability as a first-line treatment for patients with mTNBC. Patients without liver metastasis exhibited higher response rates and longer PFS than those with liver metastasis. Pretreatment IL-1α might serve as a potential biomarker for predicting PFS, while close monitoring of serum IGFBP-1, IL-2, VEGF-A and bevacizumab concentration could aid in identifying resistance.

## supplementary material

10.1136/jitc-2024-011314 online supplemental file 1

10.1136/jitc-2024-011314 online supplemental file 2

10.1136/jitc-2024-011314 online supplemental file 3

10.1136/jitc-2024-011314 online supplemental file 4

10.1136/jitc-2024-011314 online supplemental file 5

10.1136/jitc-2024-011314 online supplemental file 6

10.1136/jitc-2024-011314 online supplemental file 7

## Data Availability

Data are available upon reasonable request.
