# Supplementary figures and images for: Bevacizumab, tislelizumab and nab-paclitaxel for previously untreated metastatic triple-negative breast cancer: a phase II trial
Source: J Immunother Cancer. 2025 Apr 8;13(4):e011314. doi: 10.1136/jitc-2024-011314 (PMC11979599; doi:10.1136/jitc-2024-011314)

A

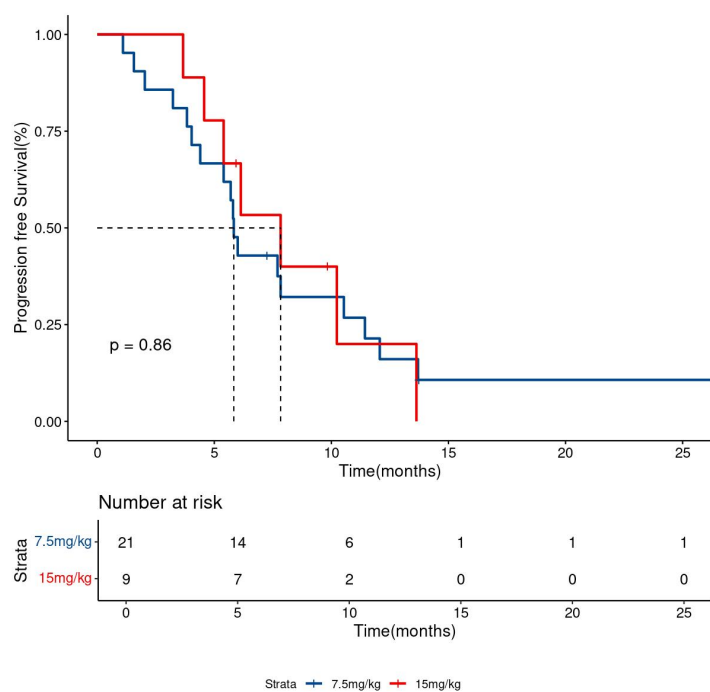

B

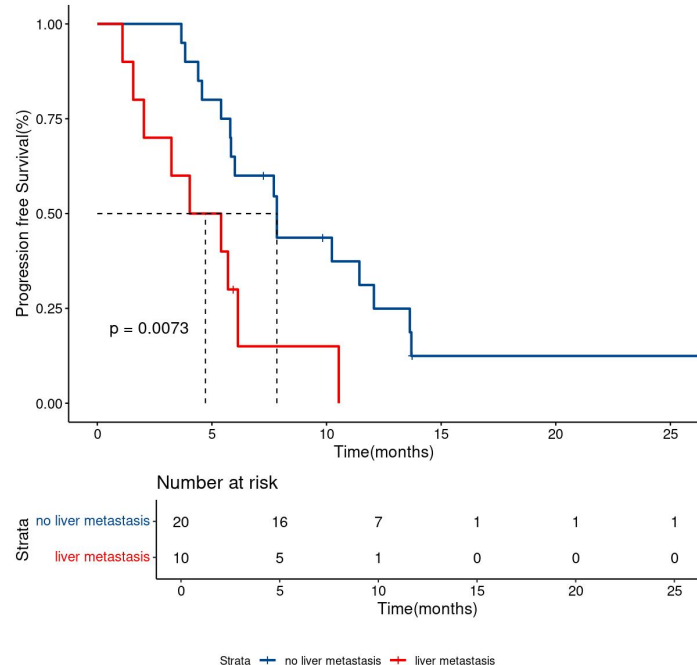

C

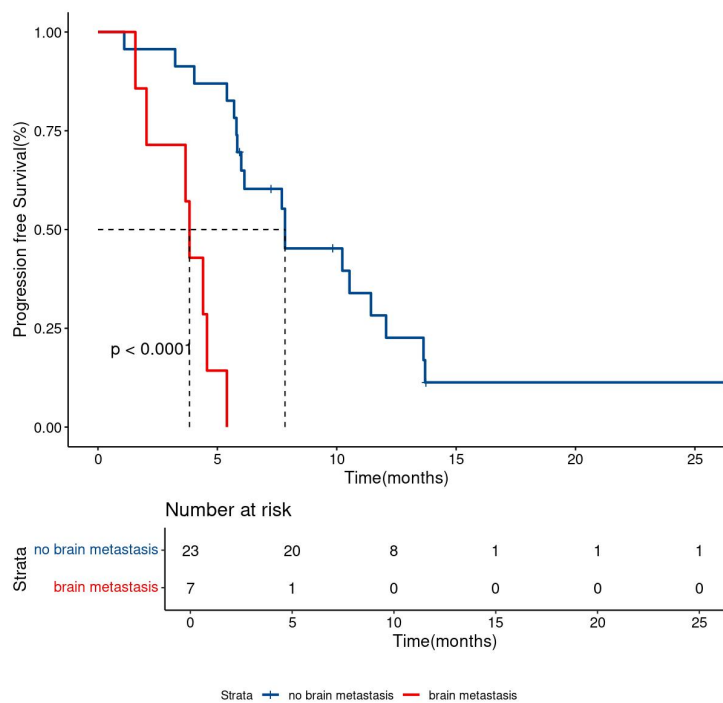

D

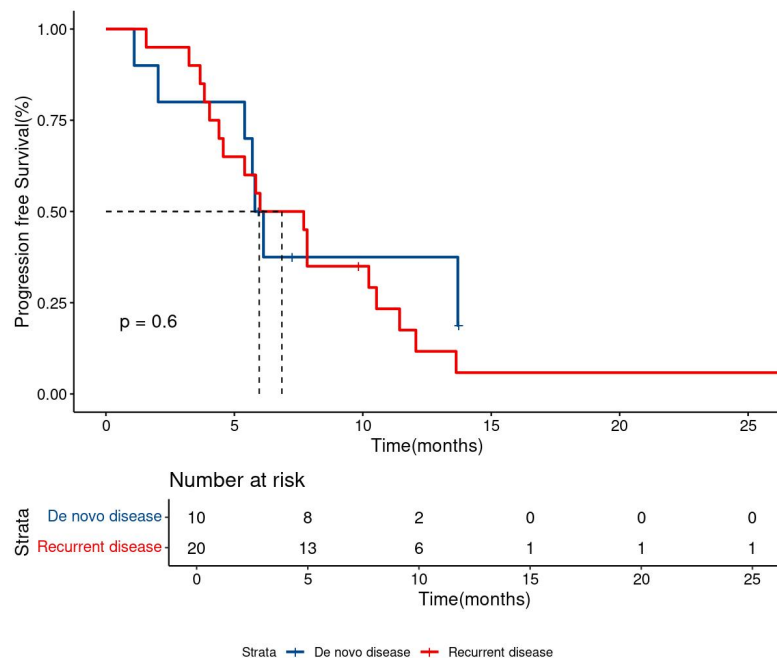

E

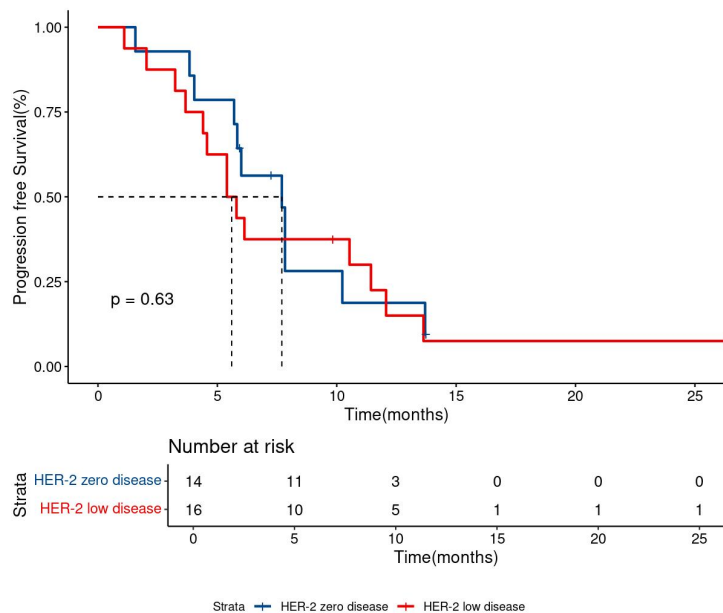

F

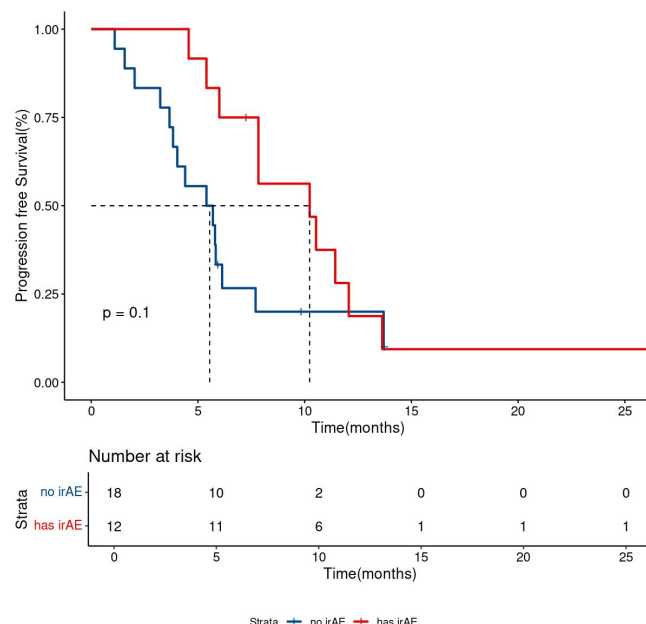

Supplement: online supplemental file 1 [file jitc-13-4-s001.pdf]

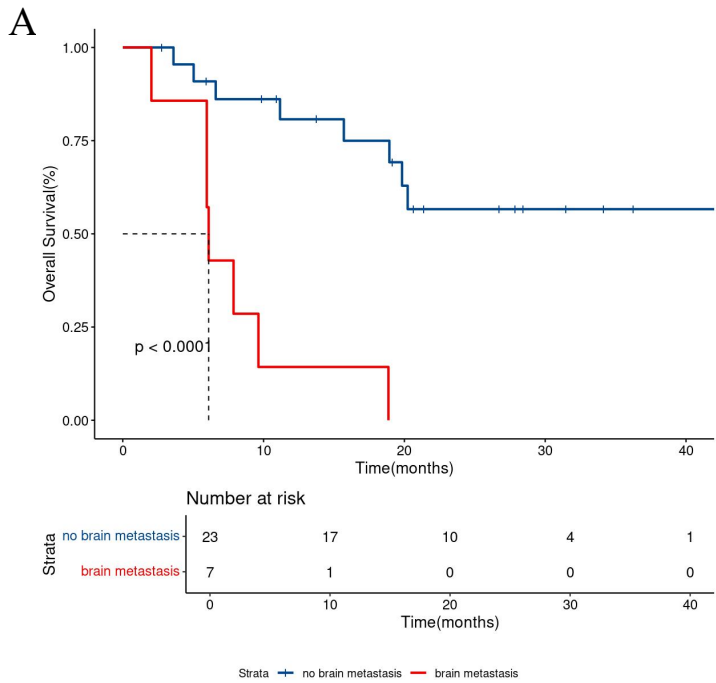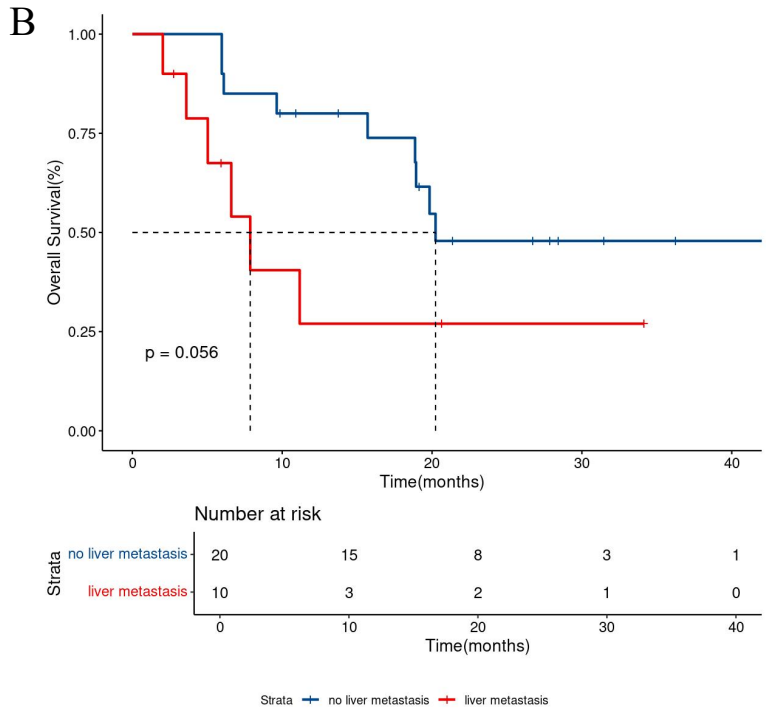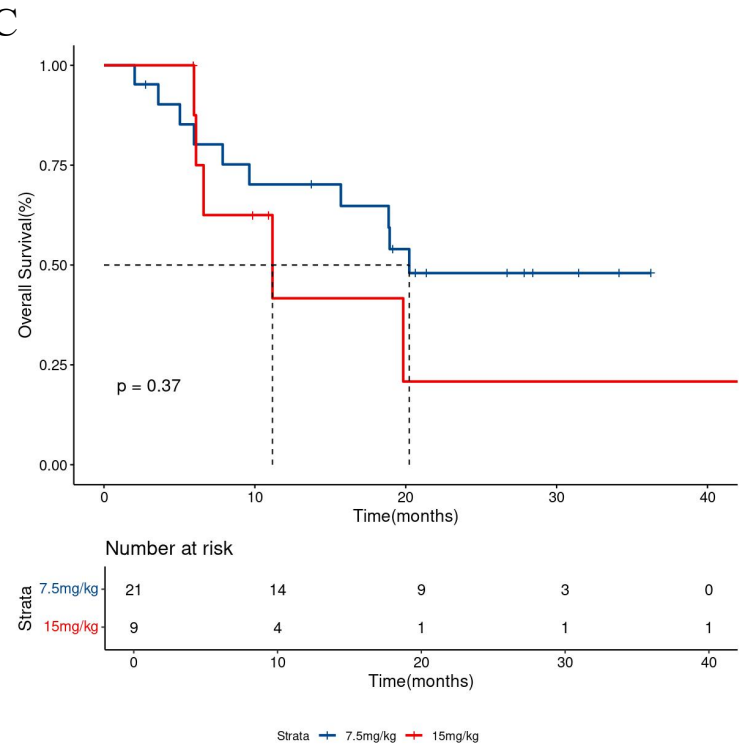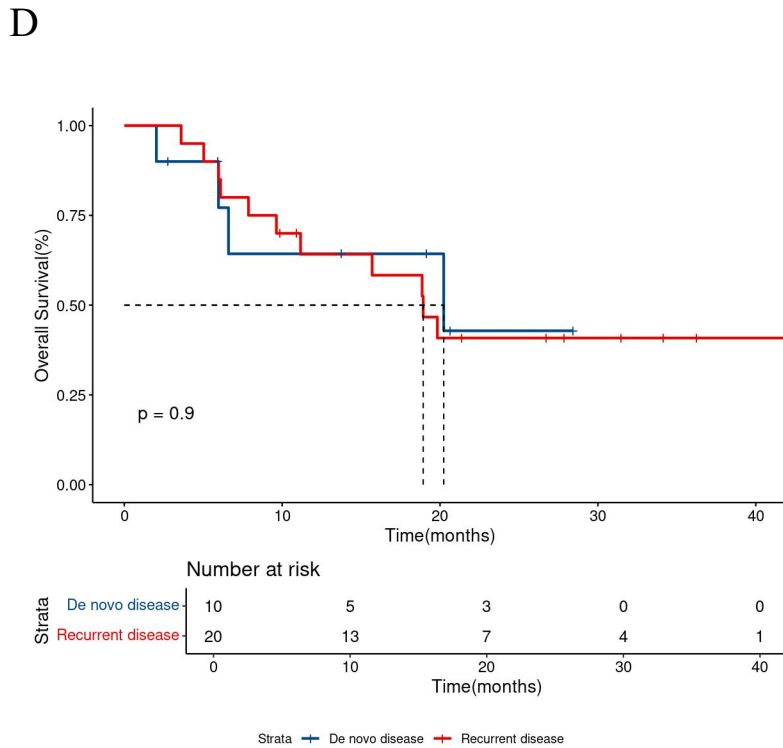

Supplement: online supplemental file 2 [file jitc-13-4-s002.pdf]

**Baseline**

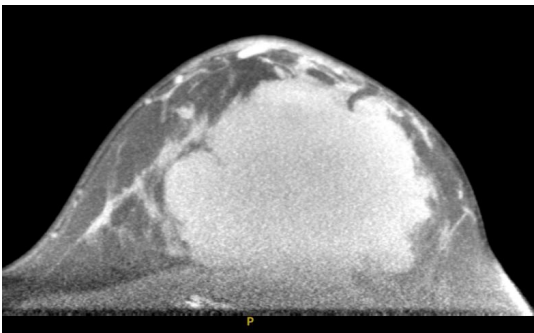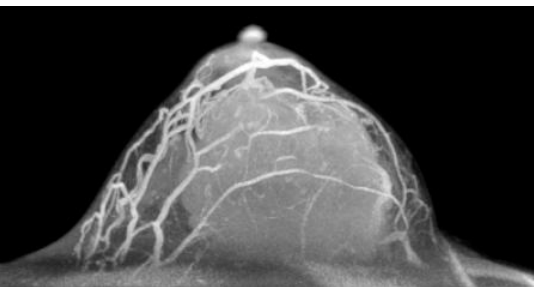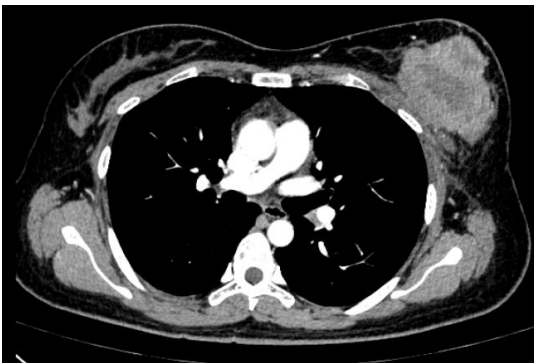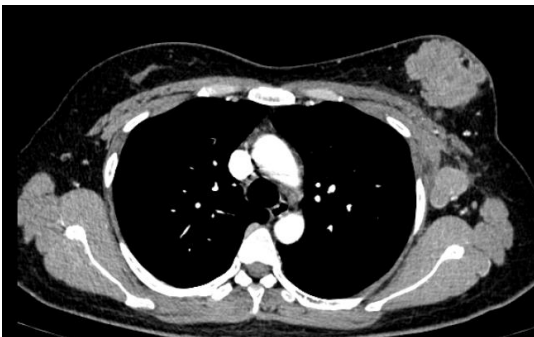

**Before surgery**

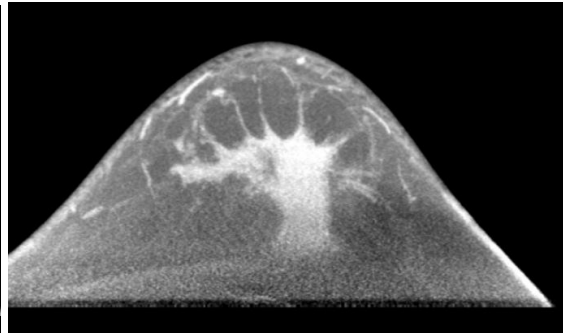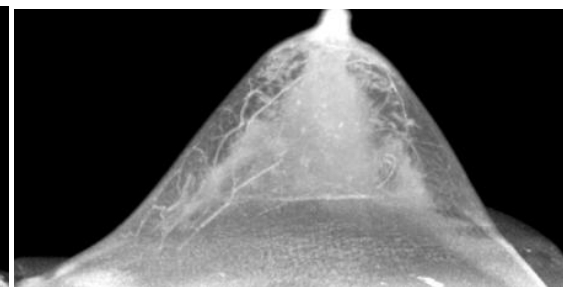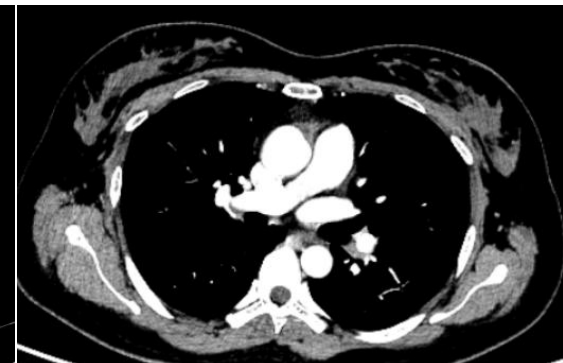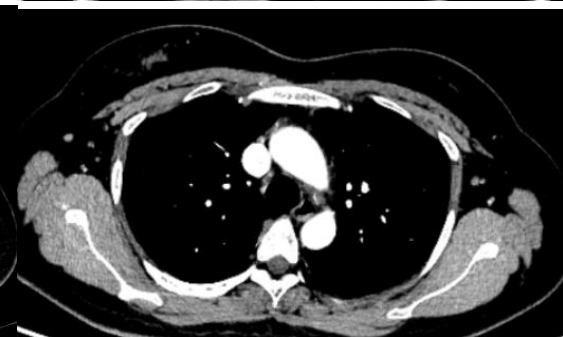

**pCR**

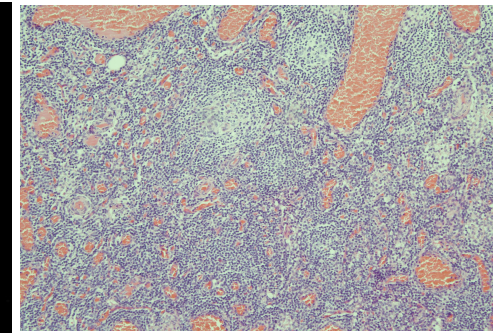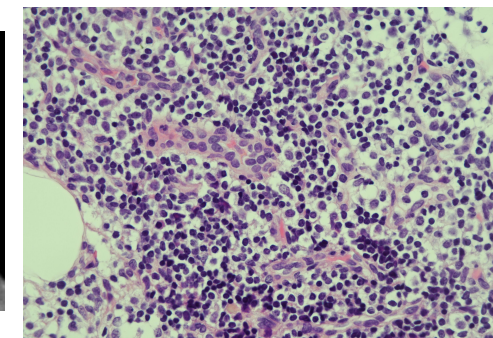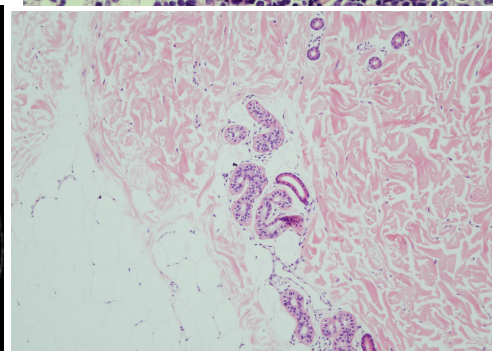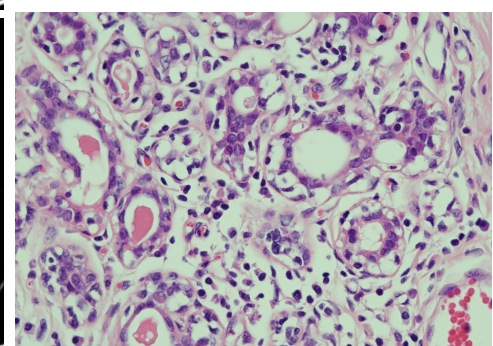

Supplement: online supplemental file 3 [file jitc-13-4-s003.pdf]

A

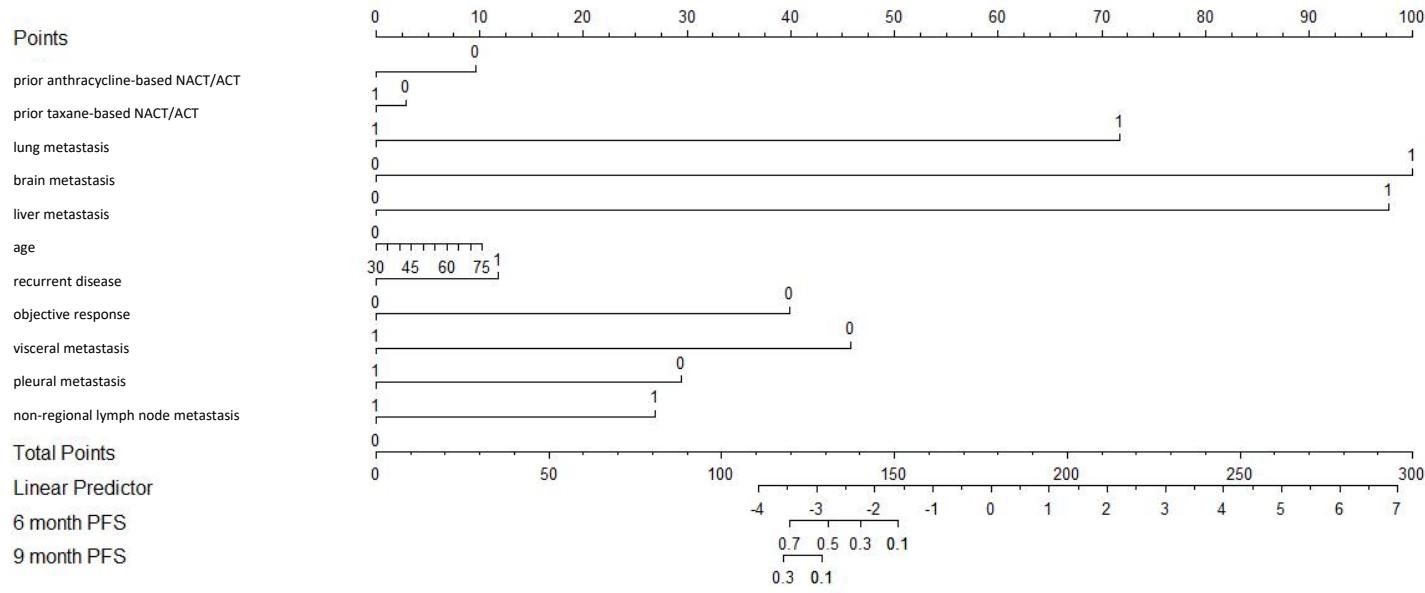

B

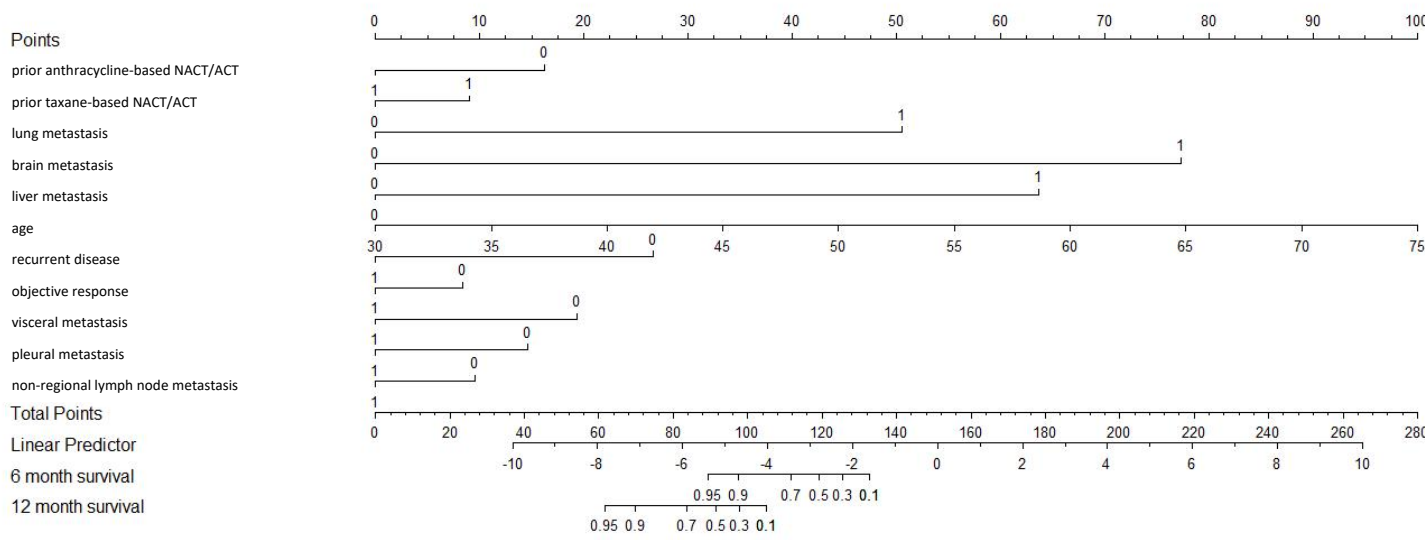

Supplement: online supplemental file 4 [file jitc-13-4-s004.pdf]

A

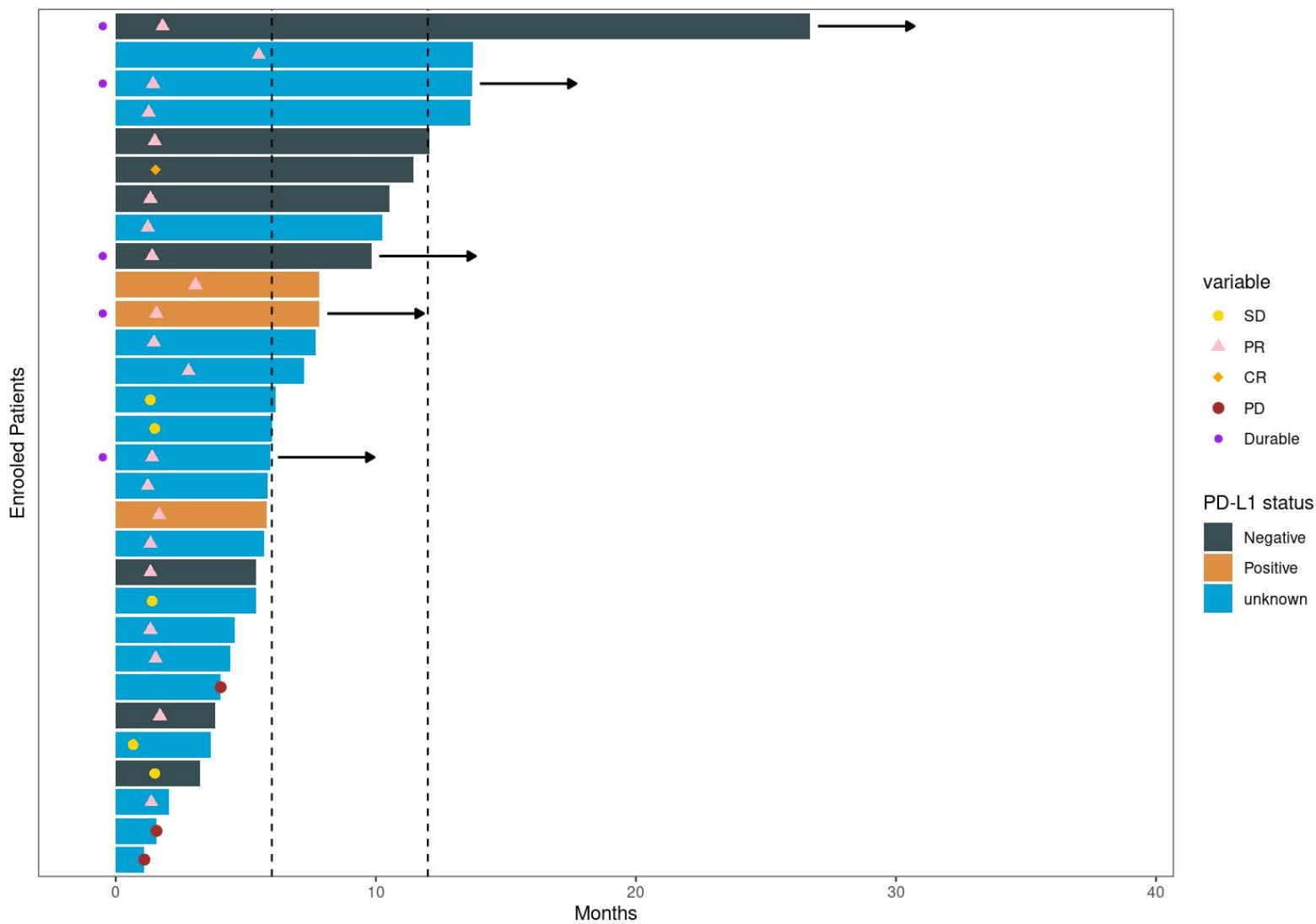

B

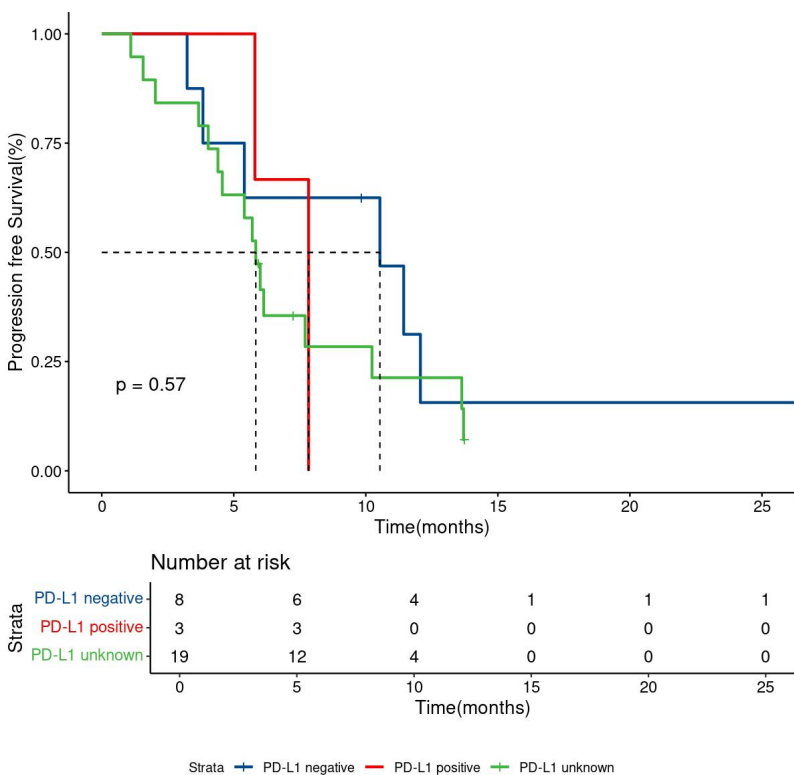

C

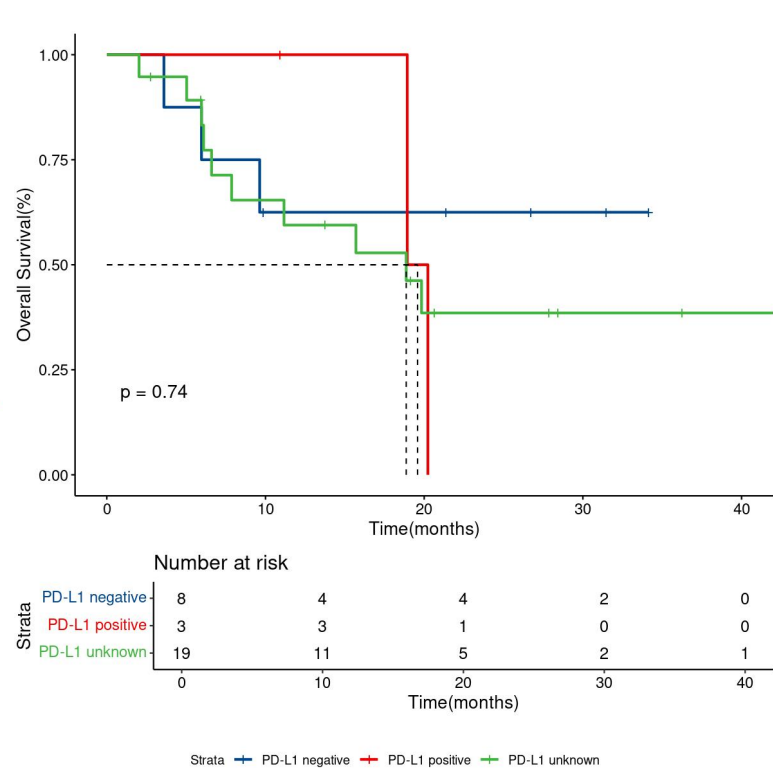

Supplement: online supplemental file 5 [file jitc-13-4-s005.pdf]

A

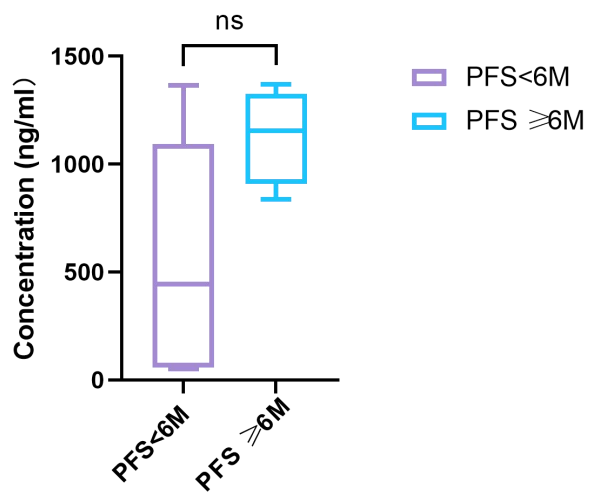

B

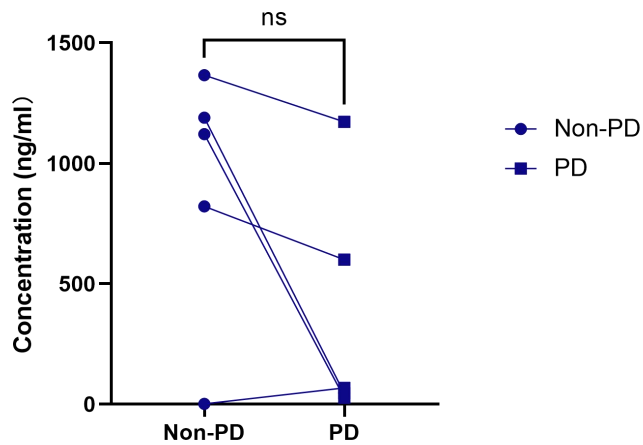

C

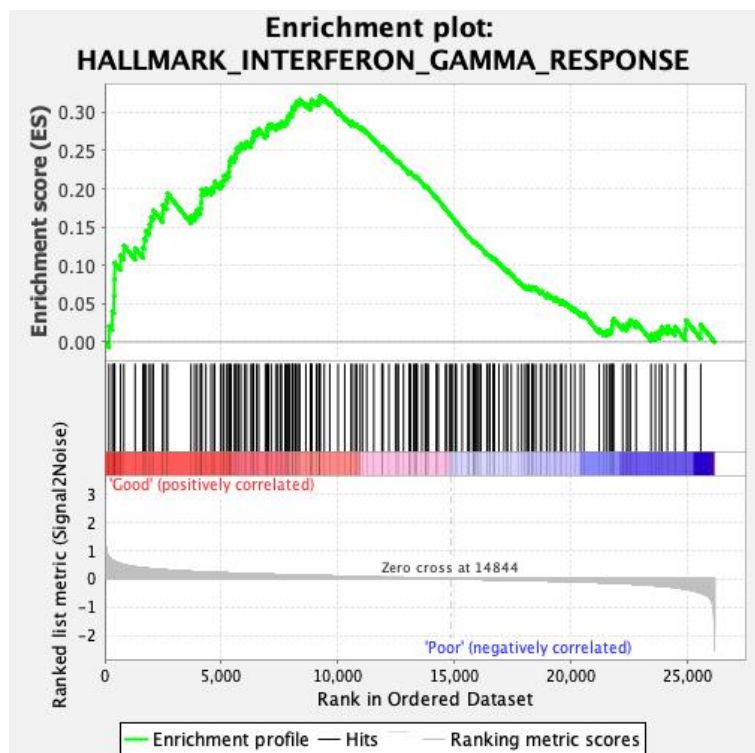

D

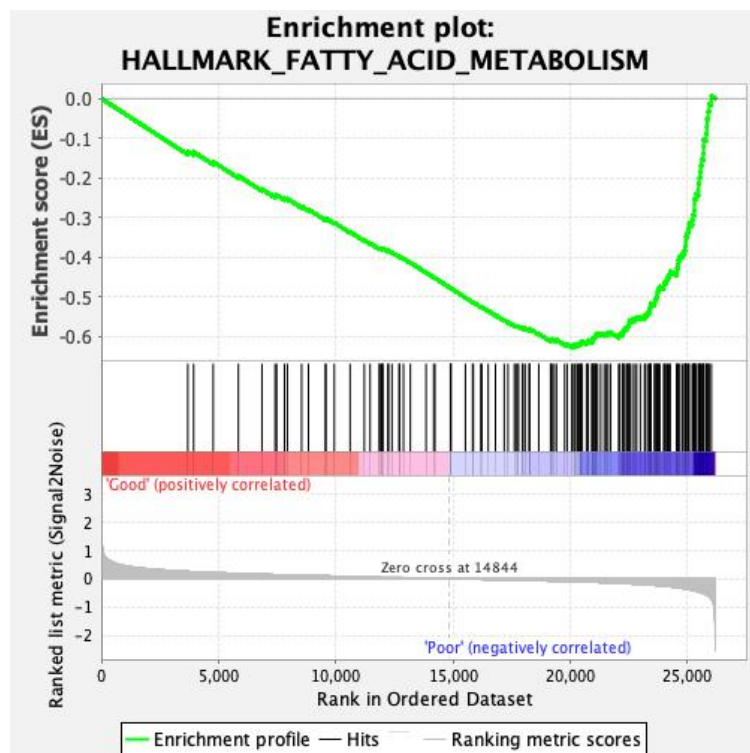

E

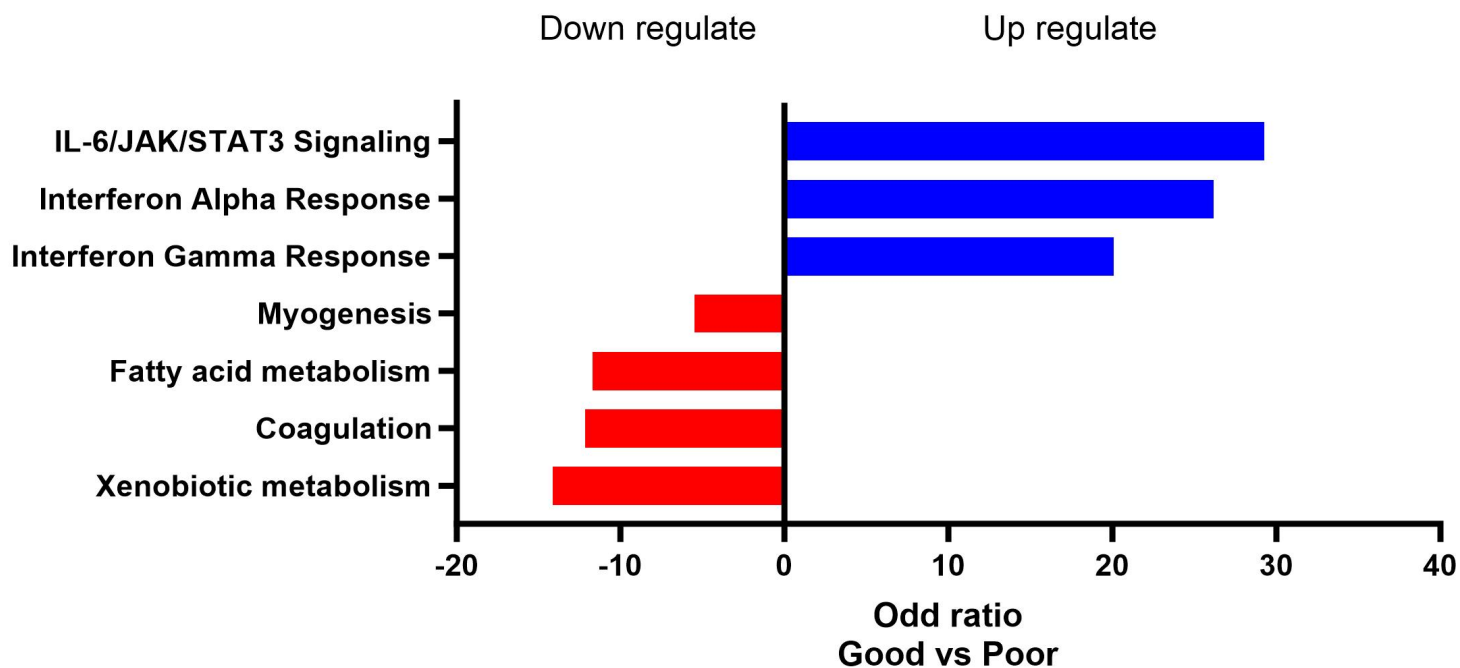

Supplement: online supplemental file 6 [file jitc-13-4-s006.pdf]
