# Supplementary material for: Bevacizumab, tislelizumab and nab-paclitaxel for previously untreated metastatic triple-negative breast cancer: a phase II trial
Source: J Immunother Cancer. 2025 Apr 8;13(4):e011314. doi: 10.1136/jitc-2024-011314 (PMC11979599; doi:10.1136/jitc-2024-011314)
Supplement: online supplemental file 7 [file jitc-13-4-s007.docx]

**Supplementary Table S1** Treatment-related adverse events in first safety evaluation

|  | **Dose of bevacizumab** | | | |  |  |
| --- | --- | --- | --- | --- | --- | --- |
|  | **7.5mg/kg**  **(N=9)** | | **15mg/kg (N=9)** | | **Overall (N=18)** | |
|  | Any Grade | Grade 3/4 | Any Grade | Grade 3/4 | Any Grade | Grade 3/4 |
| **Any AE** | 9 (100%) | 3 (33.3%) | 9 (100%) | 4 (44.4%) | 18 (100%) | 7 (38.9%) |
| **SAE** | 2 (22.2%) | 2 (22.2%) | 3 (33.3%) | 3 (33.3%) | 5 (27.8%) | 5 (27.8%) |
| **Leukopenia** | 8 (88.9%) | 2 (22.2%) | 5 (55.6%) | 1 (11.1%) | 13 (72.2%) | 3 (16.7%) |
| **Neutropenia** | 8 (88.9%) | 2 (22.2%) | 5 (55.6%) | 1 (11.1%) | 13 (72.2%) | 3 (16.7%) |
| **Febrile neutropenia** | 2 (22.2%) | 2 (22.2%) | 0 | 0 | 2 (11.1%) | 2 (11.1%) |
| **Anemia** | 9 (100%) | 0 | 5 (55.6%) | 0 | 14 (77.8%) | 0 |
| **Thrombocytopenia** | 3 (33.3%) | 1 (11.1%) | 0 | 0 | 3 (16.7%) | 1 (5.6%) |
| **Peripheral sensory neuropathy** | 7 (77.8%) | 0 | 8 (88.9%) | 0 | 15 (83.3%) | 0 |
| **Dyspepsia** | 7 (77.8%) | 0 | 6 (66.7%) | 0 | 13 (72.2%) | 0 |
| **Alopecia** | 8 (88.9%) | 0 | 5 (55.6%) | 0 | 13 (72.2%) | 0 |
| **Nausea** | 6 (66.7%) | 0 | 4 (44.4%) | 0 | 10 (55.6%) | 0 |
| **Diarrhea** | 7 (77.8%) | 0 | 2 (22.2%) | 0 | 9 (50.0%) | 0 |
| **Abdominal pain** | 6 (66.7%) | 0 | 2 (22.2%) | 0 | 8 (44.4%) | 0 |
| **Oral ulcer** | 4 (44.4%) | 0 | 5 (55.6%) | 0 | 9 (50.0%) | 0 |
| **Dizziness** | 7 (77.8%) | 0 | 2 (22.2%) | 0 | 9 (50.0%) | 0 |
| **Pruritus** | 3 (33.3%) | 0 | 4 (44.4%) | 0 | 7 (38.9%) | 0 |
| **Fatigue** | 4 (44.4%) | 0 | 3 (33.3%) | 0 | 7 (38.9%) | 0 |
| **Myalgia** | 1 (11.1%) | 0 | 3 (33.3%) | 0 | 4 (22.2%) | 0 |
| **Rash** | 1 (11.1%) | 0 | 3 (33.3%) | 0 | 4 (22.2%) | 0 |
| **Constipation** | 1 (11.1%) | 0 | 2 (22.2%) | 0 | 3 (16.7%) | 0 |
| **Arthralgia** | 1 (11.1%) | 0 | 2 (22.2%) | 0 | 3 (16.7%) | 0 |
| **Vomit** | 3 (33.3%) | 0 | 3 (33.3%) | 0 | 6 (33.3%) | 0 |
| **Hypertension** | 0 | 0 | 5 (55.5%) | 1(11.1%) | 5 (27.8%) | 1(5.6%) |
| **Epistaxis** | 1 (11.1%) | 0 | 0 | 0 | 1 (5.6%) | 0 |
| **ALT elevation** | 1 (11.1%) | 0 | 4 (44.4%) | 0 | 5 (27.8%) | 0 |
| **AST elevation** | 1 (11.1%) | 0 | 4 (44.4%) | 0 | 5 (27.8%) | 0 |
| **Serum Creatinine elevation** | 2 (22.2%) | 0 | 1 (11.1%) | 0 | 3 (16.7%) | 0 |
| **Serum total bilirubin elevation** | 1 (11.1%) | 0 | 0 | 0 | 1 (5.6%) | 0 |
| **Proteinuria** | 2 (22.2%) | 0 | 0 | 0 | 2 (11.1%) | 0 |
| **irAE** | Any Grade | Grade 3/4 | Any Grade | Grade 3/4 | Any Grade | Grade 3/4 |
| **Any irAE** | 4 (44.4%) | 1 (11.1%) | 4 (44.4%) | 1 (11.1%) | 8 (44.4%) | 2 (11.1%) |
| **Hypothyroidism** | 3 (33.3%) | **0** | 4 (44.4%) | 1 (11.1%) | 8 (44.4%) | 1 (5.6%) |
| **Hyperthyroidism** | 2 (22.2%) | **0** | 1 (11.1%) | 0 | 3 (16.7%) | 0 |
| **Myocarditis** | 1 (11.1%) | 1 (11.1%) | 0 | 0 | 1 (5.6%) | 1 (5.6%) |
| **Adrenal cortical insufficiency** | 0 | 0 | 0 | 0 | 0 | 0 |
| **Hepatitis** | 0 | 0 | 0 | 0 | 0 | 0 |
| **Rash** | 0 | 0 | 0 | 0 | 0 | 0 |

**Table S2** Univariate and multivariate analyses of potential prognostic factors for PFS.

|  | **Univariate analysis** | | | **Multivariate analysis** | | | |
| --- | --- | --- | --- | --- | --- | --- | --- |
| **Characteristics** | **HR** | **95% CI** | ***P*** | | **HR** | **95% CI** | ***P*** |
| Age | 1.00 | 0.97, 1.04 | 0.842 | | 1.02 | 0.97, 1.09 | 0.4 |
| Recurrent disease | 1.27 | 0.52, 3.11 | 0.596 | | 1.71 | 0.32, 8.97 | 0.5 |
| Menopausal | 1.51 | 0.64, 3.58 | 0.351 | | - | - | - |
| Prior anthracycline-based neoadjuvant or adjuvant therapy | 1.39 | 0.63, 3.09 | 0.414 | | - | - | - |
| Prior taxane-based neoadjuvant or adjuvant therapy | 1.23 | 0.54, 2.81 | 0.628 | | - | - | - |
| Bone metastasis | 3.98 | 1.54, 10.3 | 0.004** | | 2.09 | 0.67, 6.51 | 0.2 |
| Liver metastasis | 12.3 | 3.31, 45.9 | <0.001*** | | 19.6 | 3.66, 105 | <0.001*** |
| Brain metastasis | 3.16 | 1.30, 7.66 | 0.011* | | 41.3 | 7.00, 243 | <0.001*** |
| Lung metastasis | 1.84 | 0.82, 4.14 | 0.138 | | 3.84 | 0.57, 25.8 | 0.2 |
| HER-2 low | 1.21 | 0.54, 2.69 | 0.645 | | - | - | - |
| PD-L1 status |  |  |  | |  |  |  |
| Negative | - | - | - | | - | - | - |
| Positive | 1.73 | 0.42, 7.20 | 0.448 | | 2.35 | 0.27, 20.5 | 0.4 |
| Unknown | 1.64 | 0.63, 4.26 | 0.309 | | 1.65 | 0.46, 5.91 | 0.4 |
| Dose of bevacizumab (7.5mg/kg) | 0.99 | 0.88, 1.11 | 0.864 | | - | - | - |

HR = Hazard Ratio, CI = Confidence Interval, ***, *P*<0.001; **, *P*<0.01; *, *P*<0.05

**Table S3** Univariate and multivariate analyses of potential prognostic factors for OS.

|  | **Univariate analysis** | | | **Multivariate analysis** | | | |
| --- | --- | --- | --- | --- | --- | --- | --- |
| **Characteristics** | **HR** | **95% CI** | ***P*** | | **HR** | **95% CI** | ***P*** |
| Age | 1.03 | 0.98, 1.08 | 0.312 | | 1.20 | 1.08, 1.33 | <0.001 |
| Recurrent disease | 1.07 | 0.34, 3.38 | 0.902 | | 0.09 | 0.01, 1.15 | 0.064 |
| Menopausal | 2.49 | 0.89, 6.97 | 0.081 | | - | - | - |
| Prior anthracycline-based neoadjuvant or adjuvant therapy | 0.98 | 0.35, 2.71 | 0.967 | | - | - | - |
| Prior taxane-based neoadjuvant or adjuvant therapy | 1.07 | 0.37, 3.15 | 0.898 | | - | - | - |
| Bone metastasis | 4.14 | 1.46, 11.7 | 0.008 | | 1.68 | 0.40, 7.01 | 0.5 |
| Liver metastasis | 8.65 | 2.62, 28.6 | <0.001 | | 235 | 7.72, 7,163 | 0.002 |
| Brain metastasis | 2.66 | 0.93, 7.56 | 0.067 | | 345 | 12.2, 9,767 | <0.001 |
| Lung metastasis | 1.62 | 0.58, 4.47 | 0.355 | | 42.8 | 1.60, 1,145 | 0.025 |
| HER-2 low | 2.12 | 0.72, 6.22 | 0.171 | | - | - | - |
| PD-L1 status |  |  |  | |  |  |  |
| Negative |  |  |  | | - | - | - |
| Positive | 1.56 | 0.26, 9.37 | 0.627 | | 1.76 | 0.13, 24.6 | 0.7 |
| Unknown | 1.65 | 0.45, 6.02 | 0.448 | | 0.98 | 0.19, 5.03 | >0.9 |
| Dose of bevacizumab (7.5mg/kg) | 1.07 | 0.92, 1.24 | 0.371 | | - | - | - |

HR = Hazard Ratio, CI = Confidence Interval, ***, *P*<0.001; **, *P*<0.01; *, *P*<0.05
